# An Adaptive Sinusoidal-Disturbance-Strategy Sparrow Search Algorithm and Its Application

**DOI:** 10.3390/s22228787

**Published:** 2022-11-14

**Authors:** Feng Zheng, Gang Liu

**Affiliations:** School of Information and Communication Engineering, Beijing University of Posts and Telecommunications, Beijing 100876, China

**Keywords:** adaptive sinusoidal disturbance, population quality, adaptive Cauchy mutation, LSTM neural network, passenger flow prediction

## Abstract

In light of the problems of slow convergence speed, insufficient optimization accuracy and easy falling into local optima in the sparrow search algorithm, this paper proposes an adaptive sinusoidal-disturbance-strategy sparrow search algorithm (ASDSSA) and its mathematical equation. Firstly, the initial population quality of the algorithm is improved by fusing cubic chaos mapping and perturbation compensation factors; secondly, the sinusoidal-disturbance-strategy is introduced to update the mathematical equation of the discoverer’s position to improve the information exchange ability of the population and the global search performance of the algorithm; finally, the adaptive Cauchy mutation strategy is used to improve the ability of the algorithm to jump out of the local optimal solutions. Through the optimization experiments on eight benchmark functions and CEC2017 test functions, as well as the Wilcoxon rank-sum test and time complexity analysis, the results show that the improved algorithm has better optimization performance and convergence efficiency. Further, the improved algorithm was applied to optimize the parameters of the long short term memory network (LSTM) model for passenger flow prediction on selected metro passenger flow datasets. The effectiveness and feasibility of the improved algorithm were verified by experiments.

## 1. Introduction

**Figure 1:**
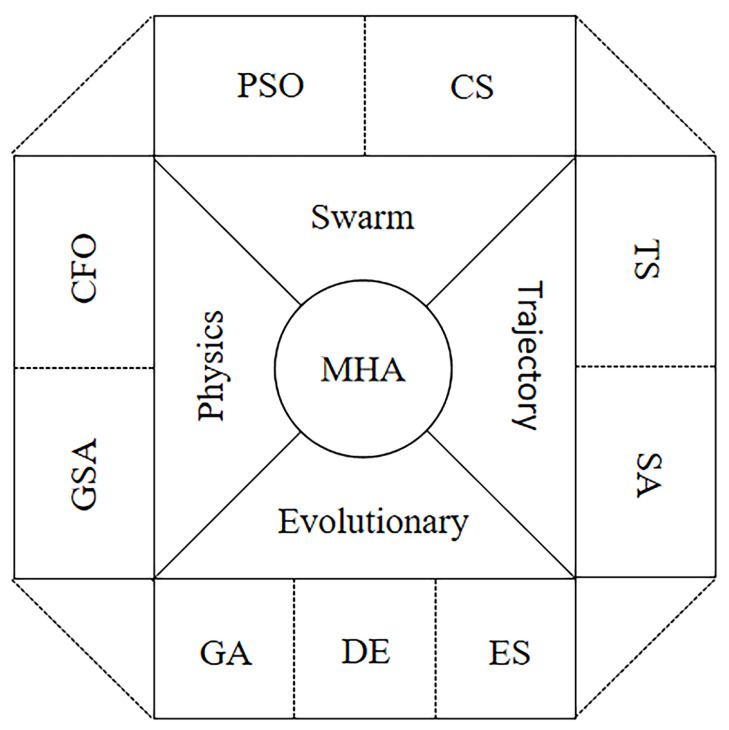
Classification of metaheuristic algorithms.

In recent years, more and more metaheuristic algorithms (MHAs) have been developed and applied widely. Successful metaheuristic algorithms improve the ability to search for optimal solutions in the search region. MHAs are broadly classified into four main categories [1,2], including evolutionary algorithms (EAs), trajectory-based algorithms (TBAs), swarm-based algorithms (SBAs) and physics-based metaheuristics (PBAs) (see Figure 1). EAs are algorithms based on the evolution of species, including the genetic algorithm (GA) [3], differential evolution (DE) [4], evolution strategy (ES) [5], etc. Inspired by biology, TBAs evolve a single trajectory of search points, including tabu search (TS) [6], simulated annealing (SA) [7], etc. SBAs are called swarm intelligence optimization algorithms, which are derived from swarm intelligence, including particle swarm optimization (PSO) [8], cuckoo search (CS) [9], etc. PBAs are derived from the existing laws of physics, including gravitational search algorithm (GSA) [10], central force optimization (CFO) [11], etc.

The swarm intelligence optimization algorithm is a stochastic search algorithm developed inspired by the social behavior patterns, evolutionary mechanisms and physical phenomena of natural biological groups. It solves the problems of traditional optimization algorithms to optimize complex problems with high computation, high complexity, low efficiency, etc. In 2011, Krishnanand K N et al. [12] proposed a firefly algorithm (FA) inspired by fireflies’ information exchange when searching for food and mating through flickering. In 2012, Pan W T et al. [13] proposed the fruit fly optimization algorithm (FOA) inspired by fruit flies’ foraging. Liu C et al. [14] proposed the wolf pack algorithm (WPA) that mimics the behaviors of bionic wolves, such as wandering, tracking, rounding up and remembering. In 2013 and 2014, Tang R et al. [15], Fong S et al. [16] and Mirjalili S et al. [17] proposed a wolf pack search algorithm (WPSA) and a gray wolf algorithm (GWO). In 2014, Meng X et al. [18] proposed chicken swarm optimization (CSO) that simulates the chicken flock hierarchy and foraging behavior. In 2015, Uymaz S A et al. [19] proposed the artificial algae algorithm (AAA) based on the living behavior of photosynthetic species of microalgae. Mirjalili S et al. [20] proposed the ant lion optimizer algorithm (ALO) based on ants, antlions and elite antlions representing different roles and constantly searching around the local optimal solution in the iterative process. In 2016, Mirjalili et al. [21] proposed the whale algorithm (WOA) stemming from the simulation of hunting behavior of humpback whale populations in nature. Mashwani W K et al. [22] introduced the latest decomposition and indicator function-based evolutionary algorithms for multi-objective optimization processing that can be used as an adaptation evaluation technique for metaheuristic algorithms. In 2017, Mirjalili S et al. [23] proposed a salp swarm algorithm (SSA) inspired by salp aggregation predation behavior. In 2018, Arora S et al. [24] proposed the butterfly optimization algorithm (BOA) inspired by butterfly positioning food predation. In 2020, Xue and Shen proposed the sparrow search algorithm (SSA) [25] based on a study of the predation and anti-predation behavior of the sparrow population. In 2021, Mashwani W K et al. [26] proposed a multiswarm-intelligence-based algorithm (MSIA) based on the single optimization problem with bounded constraints. Mashwani W K et al. [27] proposed a hybrid TLBO (HTLBO) based on a set of optimization solutions provided in a single simulation, aiming to further improve the exploration and development capabilities of the baseline TLBO algorithm [28]. Mashwani W K et al. [29] proposed an improved evolutionary algorithm integrating strategies based on large-scale global optimization problems.

Currently, swarm intelligence optimization algorithms such as sparrow intelligence are widely used for engineering problems. Adrián Rodríguez-Ramos et al. [30] used a difference algorithm and particle swarm optimization to adjust the kernel parameters in the fuzzy C-means (KFCM) algorithm for fault detection, and Yang et al. [31] used the bat algorithm (BA) to solve engineering optimization tasks. However, SSA has the advantages of strong optimization, less adjustment parameters and strong robustness, which is widely used in image analysis [32], track planning [33] and power [34].

However, when SSA calculates the initial value of fitness, the population members are randomly generated within the specified search range, which results in low distribution uniformity of the initial population within the search range. It is easy to fall into a local optimal solution in the iterative process, which greatly affects the optimization performance of the algorithm. In response to this shortcoming, many scholars have carried out research to improve the situation. Tang et al. [35] cross-integrated the bird swarm algorithm and genetic algorithm into the sparrow search algorithm, avoiding the disadvantage of easily falling into local optima in the late iterations. Chen et al. [36] initialized the sparrow population’s location by Tent chaotic mapping and combined levy flight and random wandering strategies to enhance the convergence speed and exploration ability of the algorithm. Ouyang et al. [37] used K-means clustering method to cluster and differentiate the individual locations of sparrows, thereby improving the initial quality of the population. Zhang et al. [38] used logistic mapping to initialize the population and used the Cauchy mutation operator to perturb the optimal sparrow individuals to improve the global search capability of the algorithm. Yuan et al. [39] used the centroid adversarial learning method to initialize the population of the sparrow search algorithm and introduced a mutation operator to change the position of discoverer to improve the global search efficiency of the algorithm. Zhu et al. [40] proposed an adaptive sparrow search algorithm to improve the convergence speed of SSA by introducing an adaptive learning factor. Zhang et al. [41] introduced the positive cosine algorithm into the sparrow search algorithm and proposed a new labor collaboration structure to improve the ability of the algorithm to jump out of the local optima and increase the robustness of the algorithm. Mao et al. [42] increased the ability of the algorithm to escape easy-to-fall-into local optimal solutions by introducing a Cauchy mutation and reverse learning strategy. Fu et al. [43] introduced the elite chaotic reverse learning mechanism to improve the quality of the initial population and used the chicken swarm algorithm to optimize the followers, to enhance the global search ability of the algorithm; finally, the Cauchy-Gaussian mutation strategy is used to ensure the anti-stagnation ability of the population and avoid premature convergence of the algorithm.

Although the above improvement strategies have improved the optimization ability of SSA to a certain extent, the problems of insufficient search ability and easy premature convergence still exist in the later period of optimization. To address the limitations of SSA, this paper proposes an adaptive sinusoidal-disturbance-strategy sparrow search algorithm (ASDSSA). Our contributions in this article are mainly as follows:

(1) We integrated the cubic chaos mapping and perturbation compensation factor to initialize the population, which improves the quality and traversal of the initial sparrow population.
(2) A sinusoidal disturbance strategy is proposed to update the position of the discoverers, which improves the information exchange ability between populations and improves the global search ability of the algorithm.
(3) The adaptive Cauchy mutation strategy is used to locally disturb the optimal solution, which improves the convergence speed of the algorithm and its ability to solve the problem of easily falling into the local optimal solutions.
(4) The effectiveness of the improved algorithm was demonstrated by simulating eight benchmark test functions and CEC2017 test functions, along with a comparison with other algorithms in terms of Wilcoxon rank sum tests and time complexity analysis. We applied ASDSSA to the parameter selection of the LSTM model and further verified the effectiveness and feasibility of the improved algorithm for practical engineering on the subway passenger flow dataset.

## 2. Sparrow Search Algorithm

The sparrow search algorithm is a swarm intelligence optimization algorithm proposed to simulate the behavior of sparrows foraging and avoiding predators. In the process of foraging, sparrows are divided into discoverers and followers, and a certain proportion of sparrows will be selected in the population. When reconnaissance and early warning work, the food is abandoned in time when danger is found, and the process of the sparrow constantly searching for better food is the process to optimize.

During each iteration, the positions of discoverers are updated as follows:

(1) $$X_{i,j}^{t+1}=\left\{\begin{array}{cc} X_{i,j}^{t}\cdot \exp\left(-\frac{i}{\alpha \cdot T_{\max}}\right), & R_{2}<\mathrm{ST}\\ X_{i,j}^{t}+Q\cdot L, & R_{2}\geq \mathrm{ST} \end{array}\right.$$

In Equation (1): t is the current number of iterations; $T_{\max}$ is the maximum number of iterations; $X_{i,j}^{t}$ is the positional information of the i-th sparrow in the j-th dimension; α∈(0,1] is a random number; $R_{2}$ and ST denote the warning value and safety value, respectively; Q is a random number obeying the normal distribution; L is a unit vector. When $R_{2}<\mathrm{ST}$, it means that there is no predator around the foraging environment and the finder conducts a wide area search; when $R_{2}\geq \mathrm{ST}$ it means that some sparrows find a predator. They need to fly to other safe areas to feed.

The positions of the followers are updated as follows:

(2) $$X_{i,j}^{t+1}=\left\{\begin{array}{cc} Q\exp\left(\frac{X_{\mathrm{worst}}^{t}-X_{i,j}^{t}}{i^{2}}\right), & i>n/2\\ X_{P}^{t+1}+\left|X_{i,j}^{t}-X_{P}^{t+1}\right|A^{+}\cdot L, & i\leq n/2 \end{array}\right.$$

In Equation (2): $X_{\mathrm{worst}}$ is the current global worst position, $X_{P}^{t+1}$ is the optimal position occupied by the discovers, A is 1×d matrix whose value is randomly assigned to 1 or −1, and $A^{+}=A^{T}{\left({\mathrm{AA}}^{T}\right)}^{-1}$. When i>n/2, the i-th followers with a low fitness value do not have enough food and need to fly elsewhere to search for food. When i≤n/2 the positions of the followers will be constant.

When aware of danger, sparrow populations will take anti-predation actions. It is usually assumed that the sparrows aware of danger account for 10% to 20% of the total population, and the initial positions of these sparrows are randomly generated in the population.

The positions of the vigilantes are updated as follows:

(3) $$|X_{i,j}^{t+1}=\left\{\begin{array}{cc} X_{\mathrm{best}\;}^{t}+\beta \left|X_{i,j}^{t}-X_{\mathrm{best}\;}^{t}\right|, & f_{i}\neq f_{g}\\ X_{i,j}^{t}+K\left[\frac{\left|X_{i,j}^{t}-X_{worst}^{t}\right|}{\left(f_{i}-f_{w}\right)+\varepsilon }\right],\; & f_{i}=f_{g} \end{array}\right.$$

In Equation (3), $X_{\mathrm{best}}^{t}$ is the current global optimum; β is a step control parameter; K is random number, and K ∈[−1,1]; $f_{i}$ represents the current fitness value of the individual sparrow; $f_{g}$ represents the current global optimum; ε is defined as small constant to avoid the denominator being zero.

When $f_{i}\neq f_{g}$, it indicates that the positions of the sparrows at the edge of the group change; when $f_{i}=f_{g}$, it indicates that the sparrows in the middle of the group are aware of the danger and need to get close to other sparrows to minimize their risk of predation.

## 3. Improved Sparrow Search Algorithm

As the initial population is generated in a random way in the standard sparrow search algorithm, it will make the initial population division uneven, and the discoverers converge quickly to zero and converge to the position of the global optimum at the early stage of the search. It is difficult to obtain better population diversity by searching for the characteristics of individuals, so the search accuracy is not hard. In the iterative process of the algorithm, the problem of falling into local optimal solutions is common. Aiming at the above problems, this paper makes the following improvements.

### 3.1. Chaotic Disturbance Strategy

Chaotic sequence mappings are widely used in optimization search solution problems because of their randomness and ergodic nature [44,45]. By introducing chaotic sequence mappings to initialize the population, the initial population can be more evenly distributed, which helps the algorithm to perform global searching and improves the convergence speed and accuracy of the algorithm. Among the many chaotic mappings, cubic chaotic mappings have better uniform distribution performance in the range of [0,1], and their mathematical model is:

(4) $$y_{i+1}=4y_{i}^{3}-3y_{i},-1\leq y_{i}\leq 1,i=1,2,\ldots ,N,y_{0}\neq 0$$

where $y_{i}$ is a cubic sequence. If $X_{i}\in \left[lb,ub\right]$, lb and ub are the upper and lower bounds of the solution space, respectively. The cubic sequence is mapped onto individual sparrows according to Equation (5).

(5) $$X_{i}=lb+\left(ub-lb\right)\cdot \left(\frac{y_{i}+1}{2}\right)$$

In order to further improve the performance of the sparrow search algorithm, we add a perturbation compensation factor to the initialized position of the sparrow population position, which can better evaluate the feasible solution, thereby enabling the algorithm to improve population diversity and enhance the global search capability. The position update formula with the introduction of the perturbation compensation factor is:

(6) $$R_{i}=X_{i}+{\left(-1\right)}^{i}\cdot rand\left(\right)\cdot \left(ub-lb\right)$$

The population fitness value is first calculated with the introduction of the cubic chaos mapping only, and then the perturbation compensation factor is introduced and the population fitness value is recalculated. According to the fitness value, the top N sparrows with better fitness values are selected as the initial population.

### 3.2. Sinusoidal Disturbance Strategy

At the beginning of the SSA iteration, the discoverers perform a large search in the solution space region, but as shown by Equation (1), when $R_{2}$ < ST, each dimension of the discoverers decreases exponentially, leading to a decline in population diversity and easily to falling into a local optimum, causing the problem of poor search accuracy. To address this problem, this paper introduces the sinusoidal disturbance strategy. The sinusoidal disturbance strategy was introduced to the sparrow search algorithm to update the positions of the discoverers, thereby increasing the global search ability of the discoverers, which allows individuals with good fitness in the original population to be perturbed between localities at their original positions and improves the information exchange between sparrow populations. Here we propose a sinusoidal disturbance weight as follows:

(7) $$w\left(t\right)=\left[w_{\max}\cdot \left(1-\sin\left(\frac{\pi t}{2T_{\max}}\right)\right)+w_{\min}\cdot \sin\left(\frac{\pi t}{2T_{\max}}\right)\right]$$

where $w_{min}$ and $w_{max}$ are the minimum and maximum values of the weight variation range, respectively.

The discoverers’ equation improved by the sinusoidal disturbance strategy is as follows:

(8) $$X_{i,j}^{t+1}=\left\{\begin{array}{cc} X_{i,j}^{t}\cdot w\left(t\right)\cdot \exp\left(-\frac{i}{\alpha \cdot T_{\max}}\right), & R_{2}<\mathrm{ST}\\ X_{i,j}^{t}+w\left(t\right)\cdot Q\cdot L, & R_{2}\geq \mathrm{ST} \end{array}\right.$$

With the incorporation of the sinusoidal disturbance strategy, the large weight factor in the early stage of the algorithm search improves the discoverer’s ability to explore unknown regions, thereby enabling the algorithm to have a wide range of global search capabilities. In the later stage of algorithm optimization, the weight factor decreases and the local development ability of the algorithm is strong, which increases the possibility of the algorithm jumping out of the local optima and effectively improves the convergence speed of the algorithm.

### 3.3. Adaptive Cauchy Mutation Strategy

In the iterative process of SSA, the rapid assimilation of sparrow individuals easily leads to the problem of falling into the local optimal solutions. In order to solve this problem, this paper adopts the adaptive Cauchy mutation strategy, which selects the individual with the best fitness to mutate. The specific equation is as follows:

(9) $$U_{best}^{t}=X_{best}^{t}\cdot \left[1+N_{t}\cdot Cauchy\left(0,1\right)\right]$$

(10) $$N_{t}=\frac{T_{max}-t}{T_{max}}$$

In Equation (9), $U_{best}^{t}$ is the position of the individual at the optimal position after the mutation, $N_{t}$ is the mutation step, Cauchy(0,1) is the random number generated by the Cauchy distribution function, and $X_{best}^{t}$ is the individual with the current optimal fitness. From Equation (10), we can see that the mutation step $N_{t}$ adaptively decreases as the iteration number, t, increases. In the early iterations, the mutation step is larger, allowing the algorithm to jump out of the local optima, and in the late iterations, the mutation step is smaller, speeding up the convergence of the algorithm. It serves to balance the exploration and development of the optimal position of the sparrow, enabling the algorithm to jump out of the local optima.

As the position of the mutated individual sparrow is not necessarily better than the initial position, the fitness values before and after the mutation are compared. If the fitness value of the current individual is lower than the fitness value of the mutated individual, the current individual is retained. If the fitness value of the current individual is higher than the fitness value of the mutated individual, the current individual is replaced with the mutated individual. The specific equation is as follows:

(11) $$F_{\mathrm{best}\;}^{t}=\left\{\begin{array}{c} X_{\mathrm{best}\;}^{t},f\left(X_{\mathrm{best}\;}^{t}\right)<f\left(U_{\mathrm{best}\;}^{t}\right)\\ U_{\mathrm{best}\;}^{t},f\left(X_{\mathrm{best}\;}^{t}\right)\geq f\left(U_{\mathrm{best}\;}^{t}\right) \end{array}\right.$$

**Figure 2:**
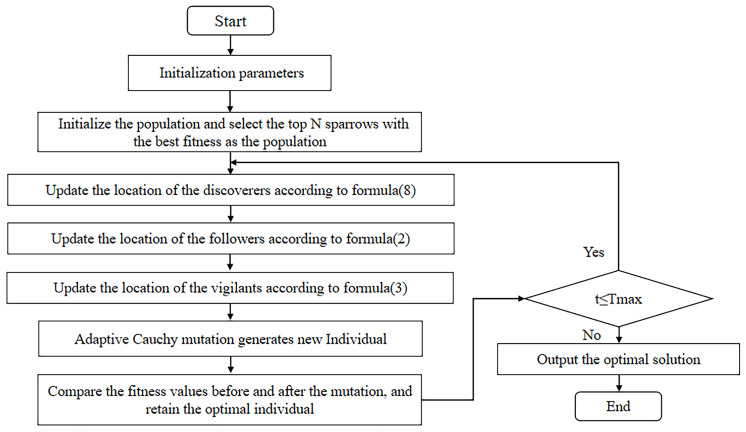
The flowchart of ASDSSA.

The corresponding ASDSSA pseudo-code is shown in Algorithm 1, and the flowchart is shown in Figure 2.
**Algorithm 1:** The framework of the ASDSSA.**Input**: Population size, N; Proportion of discoverers, PD; Proportion of vigilantes SD; Upper bounds ub; Lower bounds lb; The maximum number of iterations $T_{max}$; Weights, w;**Output**: The optimal solution, $F_{best}$; The best fitness value, $f_{g}$;1: Initialize the position of N sparrows using Equation (5), and calculating the individual sparrow fitness value $f_{i}$;2: Initialize the position of $r_{i}$ sparrows using Equation (6), and recalculating the individual sparrow fitness value;3: According to the fitness value, the top N sparrows with better fitness value are selected as the initial population;4: Get the optimal position and its corresponding optimal fitness value, the worst position and its corresponding worst fitness value;5: **while** (t < $T_{max}$)6: **for** j = 1: ND7: Update the positions of the discoverers using Equation (8);8: **end for**9: **for** j = PD: N10: Update the positions of the followers using Equation (2);11: **end for**12: **for** j = 1: SD13: Update the positions of the vigilantes using Equation (3);14: **end for**15: Selecting the best individual for the current iteration and implement the adaptive Cauchy mutation for it by Equation (9);16: If the position of the mutated individual is better than the original individual position, it will be replaced by Equation (11);17: t = t + 1;18: **end while**19: **return** $F_{best}$, $f_{g}$

### 3.4. Time Complexity Analysis

Assume that the time complexity of SSA is $O(N\times T_{max}\times d)$, where N is the population size, $T_{max}$ is the maximum number of iterations, and d is the dimensions of the problem to be optimized. Firstly, the ASDSSA is improved on the basis of the SSA, and its time complexity is divided into three parts: the time complexity of the chaotic mapping population initialization strategy with the introduction of a perturbation disturbance factor, the time complexity of the SSA with the introduction of a sinusoidal disturbance strategy and the time complexity of the SSA with the introduction of adaptive Cauchy mutation strategy. Secondly, the chaotic initialization population strategy after introducing the disturbance factor replaces the original method of randomly initializing the population, but the time complexity does not increase. The sinusoidal disturbance strategy only changes the positions of the discoverers, which can make the search range larger and more likely to include areas closer to the optimal solution, and this does not increase the time complexity. The complexity of adopting the adaptive Cauchy mutation strategy in the iterative process is $O(N\times T_{max}\times d)$ + $O(N\times T_{max}\times d)$ = $O(N\times T_{max}\times d)$. It can be seen that the time complexity of ASDSSA is consistent with the time complexity of SSA. ASDSSA is more stable, and its optimization performance is much higher than that of SSA.

## 4. Algorithmic Basic Test Functions and Analysis

### 4.1. Selection of Benchmark Test Functions

In order to verify the improvement effectiveness of the adaptive sinusoidal disturbance strategy on SSA, we adopted eight benchmark test functions with different characteristics for testing and compared our algorithm with other swarm intelligence optimization algorithms, such as WOA, GWO, PSO, GA and SSA. The parameters for each algorithm were set as shown in Table 1. The eight benchmark test functions and their specific information are given in Table 2, where $f_{1}\left(x\right)$–$f_{5}\left(x\right)$ are unimodal functions and $f_{6}\left(x\right)$–$f_{8}\left(x\right)$ are multimodal functions. The basic parameters: The maximum number of iterations: $T_{max}$ = 200; the population size: N = 30; the dimensions: dim = 30; and 30 independent runs to reduce the statistical error of the experimental results. Table 3 shows the experimental results obtained after 30 independent runs of multiple standard test functions, including the optimal solution, average value and standard deviation of each algorithm when solving each test function.

### 4.2. Experimental Results and Analysis

As can be seen in Table 3, in the experiments with the eight benchmark test functions, ASDSSA was significantly better than the other five algorithms. For the unimodal functions $f_{1}\left(x\right)$ and $f_{2}\left(x\right)$, both SSA and ASDSSA could find their theoretical optimal solutions, but the average value and standard deviation of ASDSSA were zero, indicating that ASDSSA is more stable than SSA. For the unimodal functions $f_{3}\left(x\right)$ and $f_{4}\left(x\right)$, ASDSSA found its theoretical optimal solution, and the optimal solutions of the other five algorithms were infinitely close to zero. Among them, SSA performed better, at least five orders of magnitude better than the other four algorithms. For the unimodal function $f_{5}\left(x\right)$, although the ASDSSA optimization solution did not reach the optimal value, ASDSSA had the highest optimization accuracy and the strongest stability compared with other algorithms. For the multimodal function $f_{6}\left(x\right)$, both SSA and ASDSSA could find the theoretical optimal solution, but the mean value and standard deviation of SSA were not as stable as those of ASDSSA. For multimodal functions $f_{7}\left(x\right)$ and $f_{8}\left(x\right)$, ASDSSA outperformed other algorithms in optimal value, average value and standard deviation. Comprehensive analysis showed that the average values and standard deviations of ASDSSA are better than those of other algorithms in the optimization process, indicating that the introduction of the adaptive sinusoidal disturbance strategy has improved the solution performance of SSA and provides a strong global search capability and local exploration capability.

**Figure 3:**
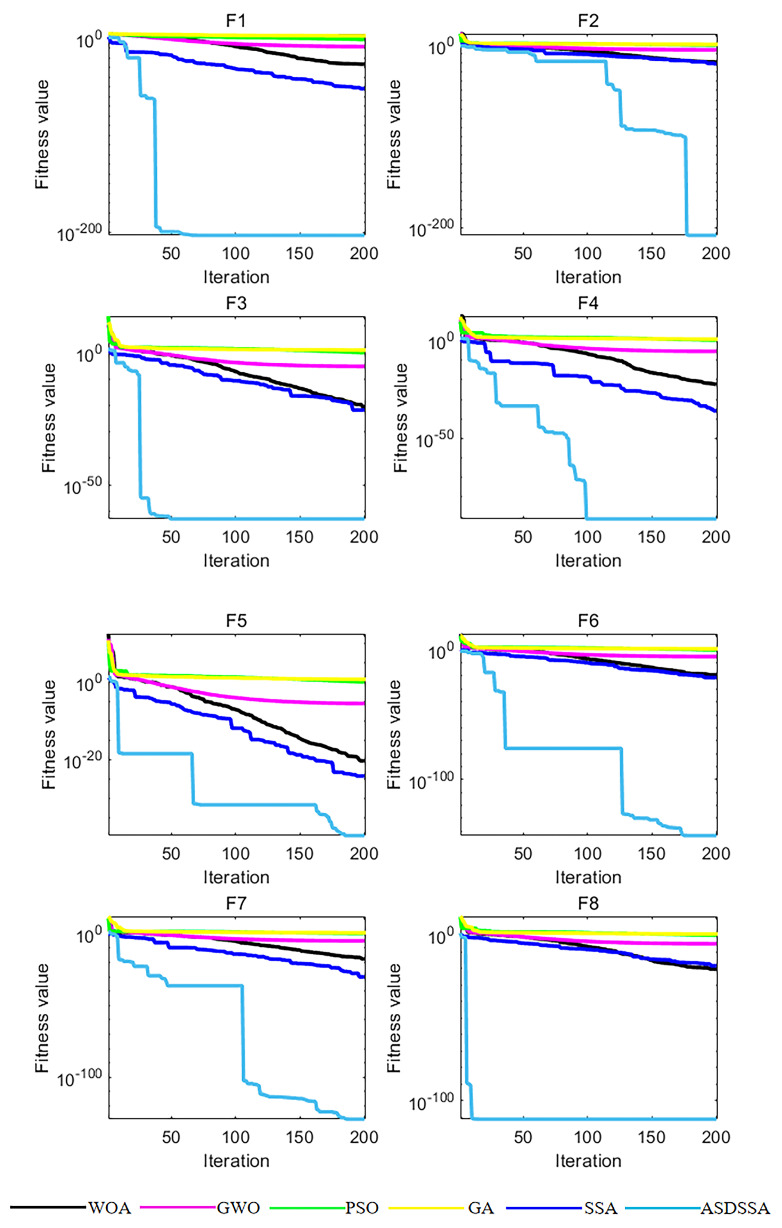
Convergence curves of test functions $f_{1}\left(x\right)$–$f_{8}\left(x\right)$.

In order to illustrate that ASDSSA has better optimization ability and convergence speed, simulation experiments were carried out on eight benchmark test functions. Figure 3 shows the convergence curves of different algorithms on the benchmark functions.

The convergence curves of all the algorithms mentioned in this paper are presented in Figure 3 for 200 iterations, where the horizontal axis represents the number of iterations and the vertical axis represents the fitness value. For all test functions, ASDSSA requires the least number of iterations to reach the peak accuracy of the others, indicating that the introduction of the adaptive sinusoidal disturbance strategy increases the proportion of high-quality individuals in the population and improves the convergence speed of the algorithm. As the number of iterations increases, the convergence curves of the GWO, PSO and GA algorithms tend to flatten out; they display varying degrees of stagnation and relatively low optimization accuracy. The step-wise decline of the ASDSSA convergence curve shows that the adaptive Cauchy mutation strategy can help the algorithm to get out of stagnation effectively and search for a better solution in the global scope. At the same number of iterations, the solution accuracy of ASDSSA is much higher than those of other algorithms. In summary, it can be proved that ASDSSA has faster convergence speed and stronger robustness than the other algorithms.

**Figure 4:**
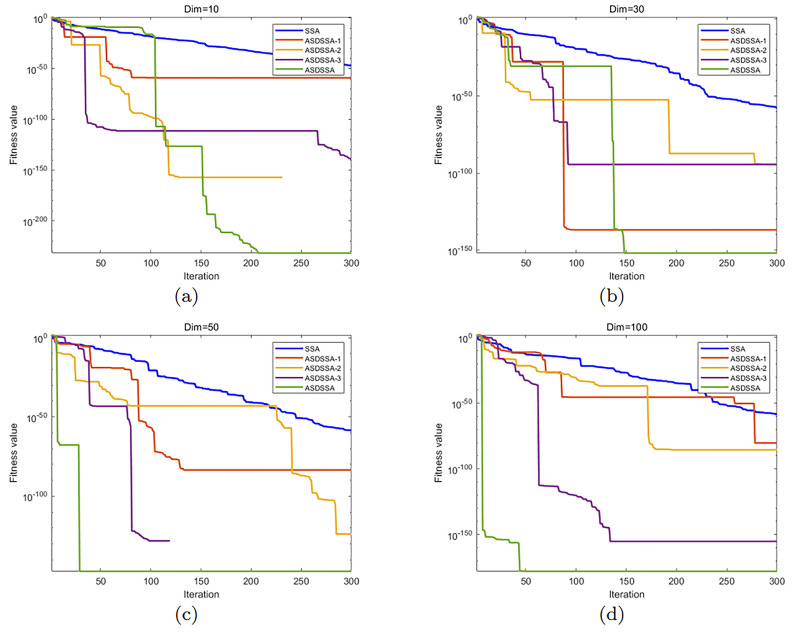
Comparison of the effectiveness of different strategies.

Figure 4 shows the comparison of the effects of the three strategies proposed in this paper. The initial population number was set to 30, the number of iterations was set to 300, and we chose $f_{1}\left(x\right)$ as the objective function; (a) is 10-dimensional, (b) is 30-dimensional, (c) is 50-dimensional and (d) is 100-dimensional. SSA is the original algorithm; ASDSSA-1, ASDSSA-2 and ASDSSA-3 are improved SSAs that include the chaotic disturbance strategy, sinusoidal disturbance strategy and adaptive Cauchy mutation strategy, respectively. As can be seen in Figure 4, the addition of any these three strategies can improve the performance of the SSA to a certain extent. In 10 dimensions, ASDSSA-1 showed no obvious improvement, but ASDSSA-2 and ASDSSA-3 were obviously superior; in 30 dimensions, ASDSSA-1 performed better than ASDSSA-2 and ASDSSA-3; in 50 dimensions, ASDSSA-2 and ASDSSA-3 performed better; in 100 dimensions, ASDSSA-1 and ASDSSA-2 performed averagely, and ASDSSA-3 performed better. In summary, ASDSSA outperformed the baseline strategy in terms of convergence speed and optimization accuracy, indicating that the population chaotic disturbance strategy, the sinusoidal disturbance strategy and the adaptive Cauchy mutation strategy all have good improvement effects on the original algorithm.

To further validate the outperformance of the ASDSSA on benchmark functions, the benchmark functions used were the sphere function, the Schwefel 2.22 function, the Ackley function, the Griewank function, the rotated hyper-ellipsoid function and the hyper-ellipsoid function. More detailed descriptions of all benchmark functions can be found in the literature [46,47]. We compared the performance of ASDSSA with that of the HSO+ algorithm [48] in 30D, 60D and 90D, which also performed well on benchmark functions. We used the same population size of 50, the same number of iterations of 50 and 100 independent runs. Table 4 gives the results of the mean and standard deviation of the ASDSSA and HSO+ algorithms on 30D, 60D and 90D dimensions. As we can see in Table 4, the optimization performance of the ASDSSA is significantly better than that of the HSO+ algorithm.

## 5. CEC2017 Test and Wilcoxon Rank-Sum Test

To further validate the performance of the ASDSSA and its ability to solve for composite type functions, we tested the ASDSSA on the CEC2017 test functions [49]. CEC2017 contains thirty test functions, of which F1–F3 are single-peaked functions, F4–F10 are simple multi-peaked functions, F11–F20 are mixed functions, F21–F30 are composite functions and the F2 was not used due to its defects. To demonstrate the superiority of the improvements in the ASDSSA, the ASDSSA is compared with other heuristic algorithms, such as WOA, GWO, PSO, SSA and the multiple-strategies sparrow search algorithm (MSSA) [42] in a CEC2017 test function optimization experiment. We set dimensions to 30, the initial population number to 100 and the maximum number of target functions to 300,000, and we performed 51 independent runs for each test function, calculating the mean and standard deviation for each algorithm based on the results, and the comparison of the data from the test function search experiments is shown in Table 5.

The results in Table 5 show that the ASDSSA produced the best mean value in F1, F3, F4, F5, F7, F8, F10, F11, F13, F17, F18, F19, F20, F21, F22, F23, F24, F27, F29 and F30, which indicates that the ASDSSA has good optimization. However, the original swarm intelligence optimization algorithms WOA, GWO, PSO and SSA performed poorly in the more complex CEC2017 test functions, indicating that the optimization ability declined in more complex optimization problems. ASDSSA outperformed the improved algorithm MSSA in mean and standard deviation, indicating that the strategy of ASDSSA is better and more suitable for complex optimization problems.

**Figure 5:**
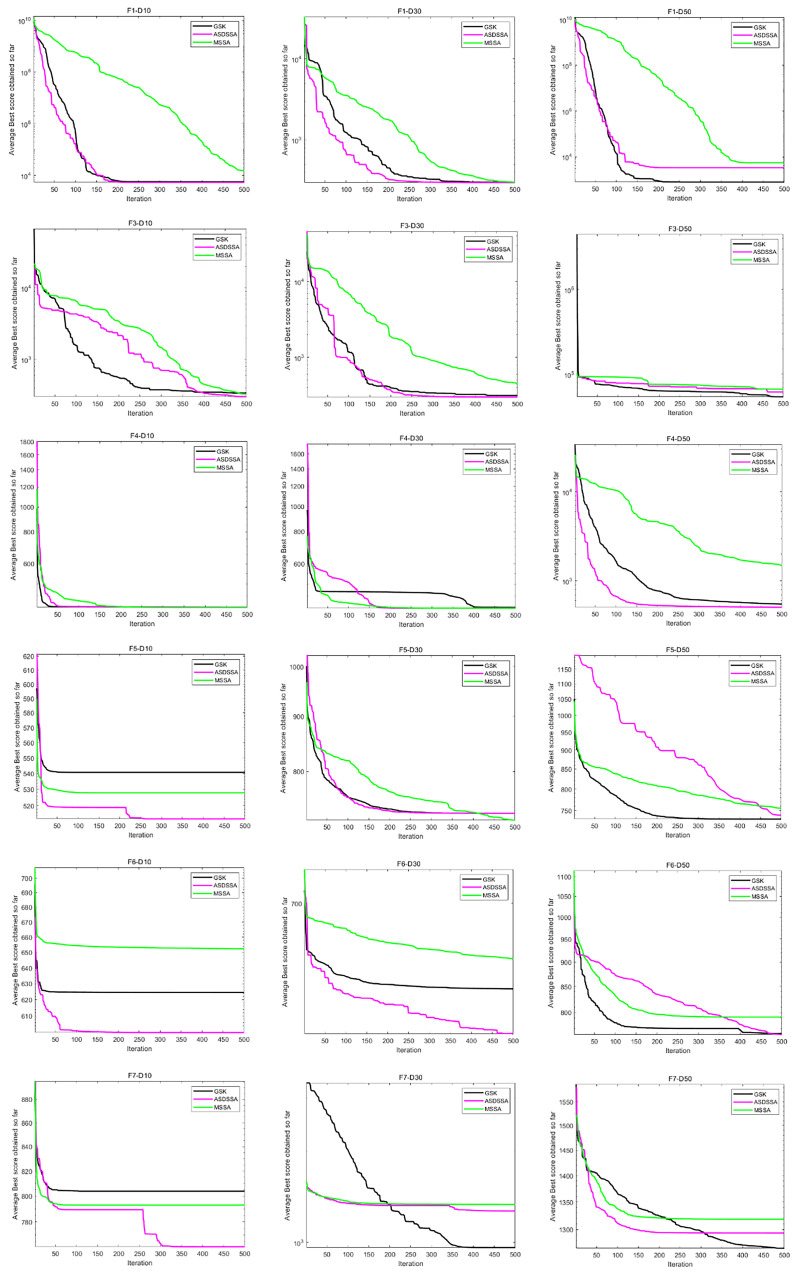
Convergence curves of F1–F7.

**Figure 6:**
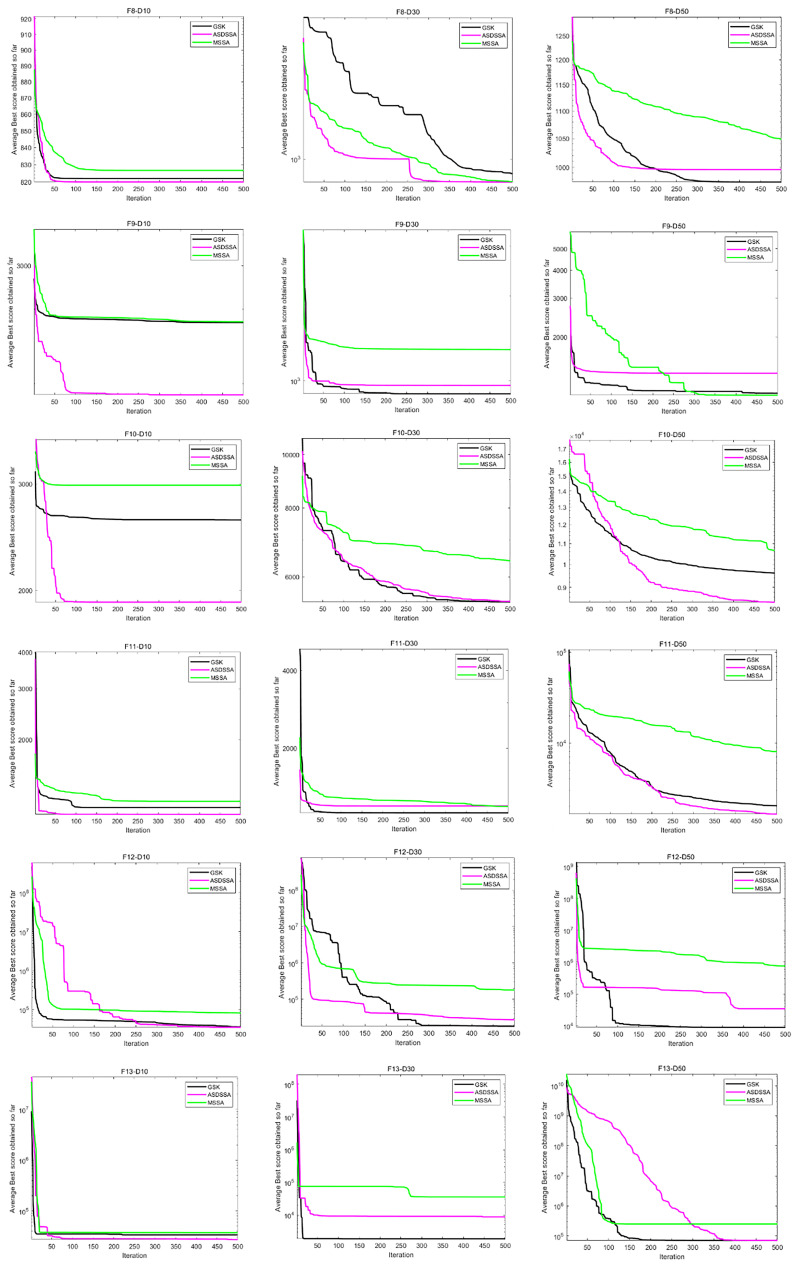
Convergence curves of F8–F13.

**Figure 7:**
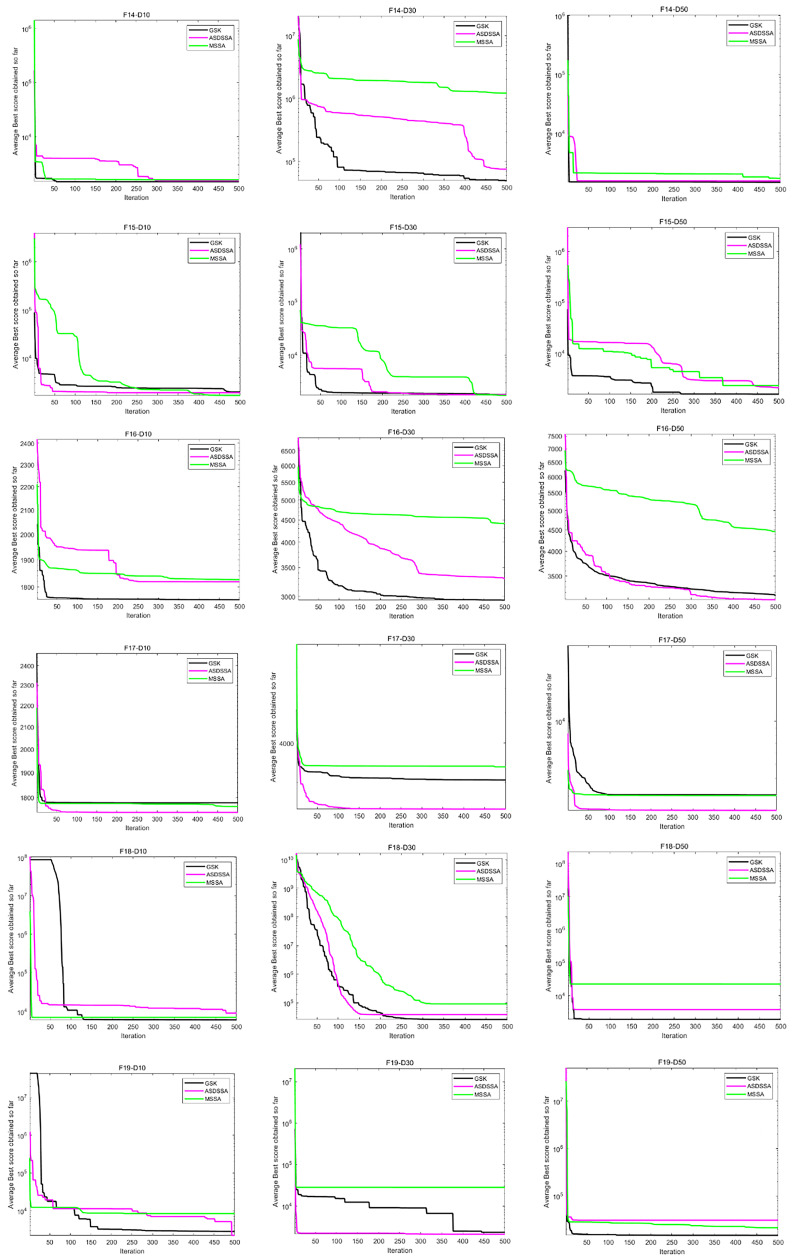
Convergence curves of F14–F19.

**Figure 8:**
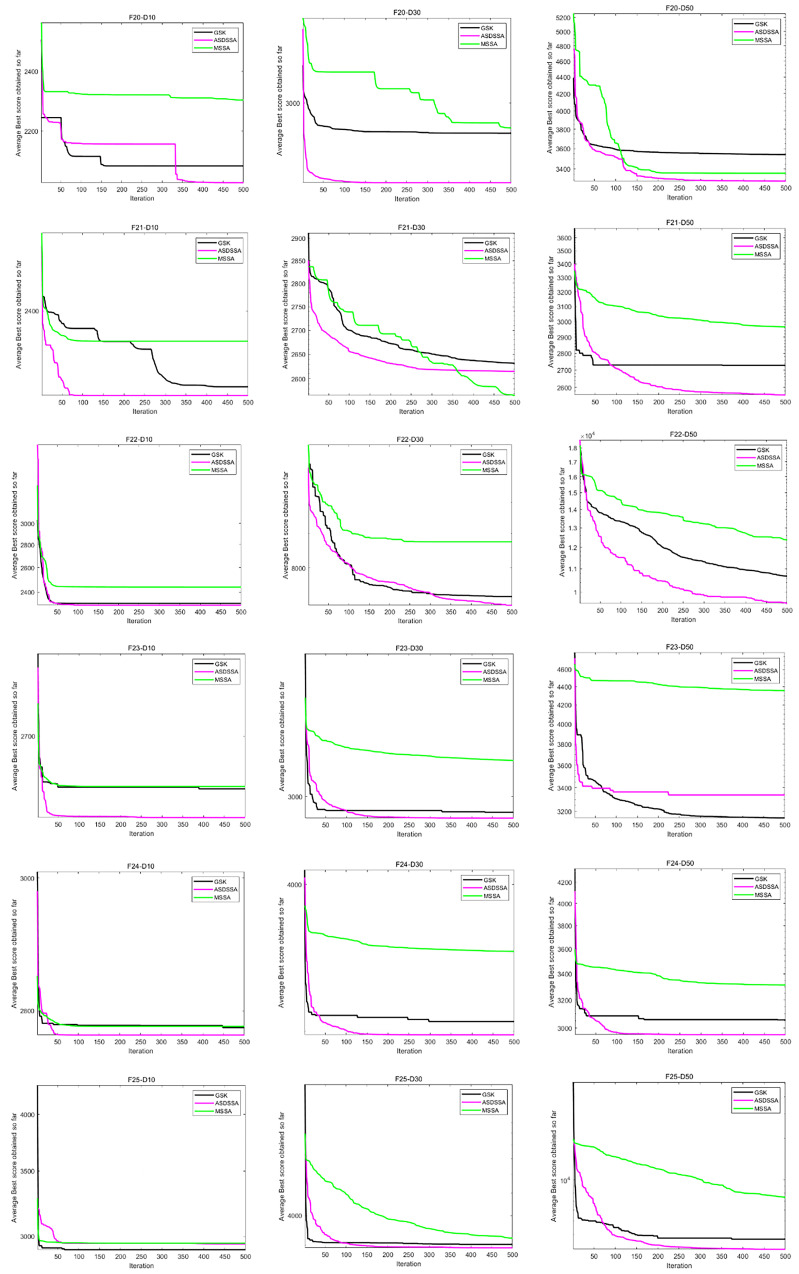
Convergence curves of F20–F25.

**Figure 9:**
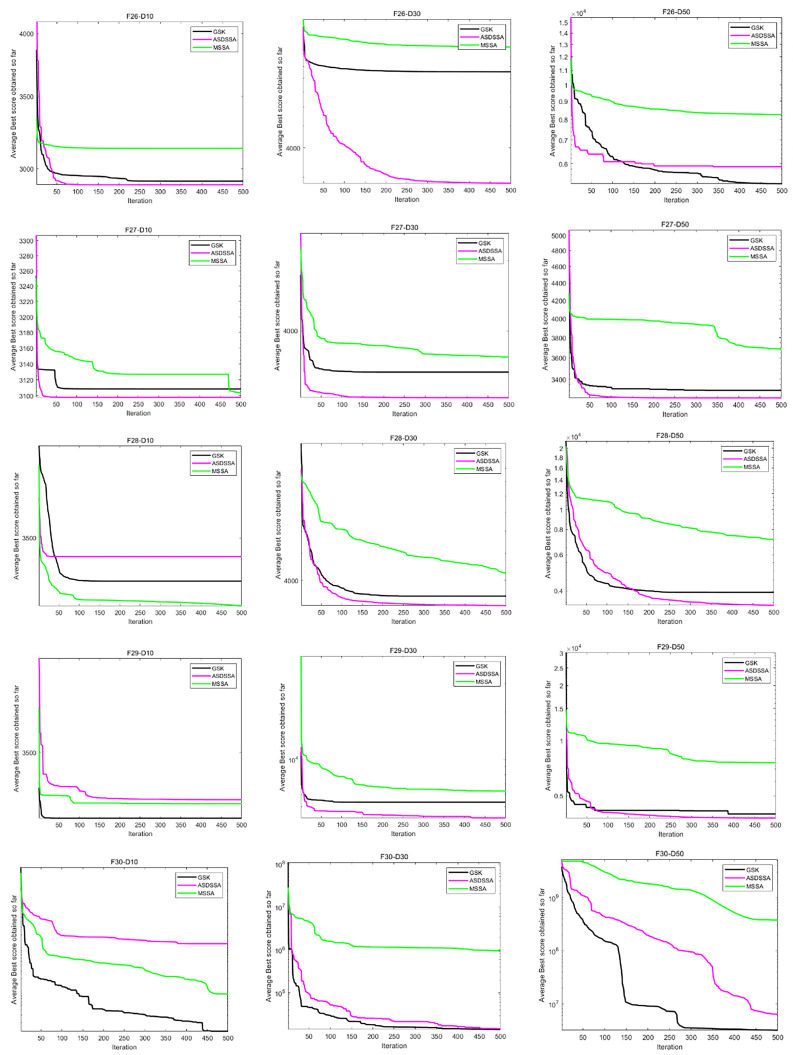
Convergence curves of F26–F30.

Similarly, the results of ASDSSA for Dim = 10, Dim = 50 and Dim = 100 are shown in Table 6. We compared the performance of ASDSSA with the gaining sharing knowledge-based algorithm (GSK) [2] using better (+), worse (−) or the statistically insignificant (≈). The statistical tests are summarized in Table 7. ASDSSA outperformed the GSK algorithm with 10, 30 and 50 dimensions, but only slightly outperformed the GSK algorithm with 100 dimensions, and overall, the ASDSSA’s performance was superior to that of the GSK algorithm. The specific convergence plots are shown below. Figure 5 shows the convergence plots for F1 to F7 solved by the proposed algorithm, the GSK algorithm and MSSA with 10, 30 and 50 dimensions. Figure 6 shows the convergence plots for F8 to F13 solved by the proposed algorithm, the GSK algorithm and MSSA for 10, 30 and 50 dimensions. Figure 7 shows the convergence plots for F14 to F19 solved by the proposed algorithm, the GSK algorithm and the MSSA with 10, 30 and 50 dimensions. Figure 8 shows the convergence plots for F20 to F25 solved by the proposed algorithm, the GSK algorithm and the MSSA with 10, 30 and 50 dimensions. Figure 9 shows the convergence plots for F26 to F30 for the benchmark functions solved by the proposed algorithm, the GSK algorithm and the MSSA with 10, 30 and 50 dimensions. According to the values and convergence plots shown, the ASDSSA possesses good properties for solving large-scale global optimization problems.

We also show a set of comparisons with the winner of the 2014 competition, LSHADE [50], for Dim = 10, Dim = 30, Dim = 50 and Dim = 100 with CEC2017. As can be seen in Table 8, the performances of the two algorithms are comparable for 10 dimensions and in 30 dimensions, but the superiority of the ASDSSA over the LSHADE algorithm increased significantly as the number of dimensions increased from 30 to 100 dimensions.

Using the results of 51 independent experiments with 30 dimensions, we used the Wilcoxon rank-sum nonparametric statistical test method [51] to test the statistically significant differences between ASDSSA and the other five algorithms to judge the reliability. The original hypothesis, H0, was that there was no significant difference between the two algorithms; hypothesis H1 was that the overall difference between the two algorithms was significant. The *p*-values of the test results were used to compare the differences between the two algorithms, and when *p* < 0.05, the two algorithms could be considered significantly different. The experimental results are indicated by “+, = and −”, where “+” indicates that by the rank-sum test, the ASDSSA is better than the comparison algorithm, “=” indicates that by the rank-sum test, the ASDSSA is comparable to the comparison algorithm and “−” indicates that by the rank-sum test, the ASDSSA is inferior to the comparison algorithm. The results of the Wilcoxon rank-sum test are shown in Table 9, which shows that the statistical results of the rank-sum tests of ASDSSA and the other algorithms are almost always less than 0.05, indicating that ASDSSA is significantly different from the other algorithms.

## 6. Application of the the Improved Sparrow Search Algorithm in Subway Passenger Flow Prediction

To further validate the optimization performance of ASDSSA and verify its ability to be applied in practical engineering, it was used to optimize the parameters of an LSTM model and applied to the short-term passenger flow forecast of a metro [52,53,54]. The LSTM model is a machine learning algorithm with a simple structure, few parameter settings, a strong generalization ability and fast learning speed. Short-term passenger flow forecasting is a basic task for the formulation of operation management and dynamic adjustment of rail transit. Accurately predicted passenger flow is very important for the operation of urban subway systems and ensures the operational safety of rail transit. As the urban rail transit data are non-linear and non-cyclical, the LSTM prediction model has been widely used, which can timely grasp the regular characteristics of short term passenger flow and always performs well.

The performance of LSTM models is closely related to the selection of parameters, and inappropriate parameter selection can reduce the learning ability and generalization performance of the algorithm. To improve the model’s performance, we used ASDSSA to optimize the learning rate, number of neurons and other parameters in the LSTM.

In this study, the automatic fare collection (AFC) data of Fuzhou urban rail transit system in December 2020 were selected as the research object, which mainly include AFC code, transaction time (exit time), entry time, entry station, exit station, card number, card type and transaction status. The passenger flow data of the first three weeks of December were used as training data, and the passenger flow data of the last week of December were used as test data for short-time passenger flow prediction. By analyzing the passenger flow data to predict the peaks and trend of passenger flow, we could formulate a suitable subway operation scheme for the short-term fluctuations of passenger flow, so as to improve economic benefits and reduce unnecessary operation losses.

In the experiment, the original data were preprocessed.The dataset was first normalized by using Equation (12) and divided into a training set and a test set; then, ASDSSA was used to optimize the parameters of the LSTM model to obtain the optimized model ASDSSA-LSTM; and finally, the prediction results were obtained through the optimized model.

(12) $$X^{*}=\frac{X-X_{\min}}{X_{\max}-X_{\min}}$$

In order to verify the accuracy and generalization effect of the optimized model, we constructed BP, LSTM, WOA-LSTM, GWO-LSTM, PSO-LSTM, SSA-LSTM, MSSA-LSTM and ASDSSA-LSTM—eight models for comparison. Mean absolute percent error (MAPE), root mean-square error (RMSE), mean absolute error (MAE) and coefficient of determination (R-squared, $R^{2}$) were used as evaluation metrics to validate the effectiveness of the prediction model. They were calculated as follows:

(13) $$\mathrm{MAPE}=\frac{1}{\;m}\sum_{i=1}^{m}\left|\frac{{\hat{y}}_{i}-y_{i}}{y_{i}}\right|$$

(14) $$\mathrm{MAE}=\frac{1}{\;m}\sum_{i=1}^{m}\left|{\hat{y}}_{i}-y_{i}\right|$$

(15) $$\mathrm{RMSE}=\sqrt{\frac{1}{m}\sum_{i=1}^{m}{\left({\hat{y}}_{i}-y_{i}\right)}^{2}}$$

(16) $$R^{2}=1-\frac{\sum_{i=1}^{m}{\left({\hat{y}}_{i}-y\right)}^{2}}{\sum_{i=1}^{m}{\left({\bar{y}}_{i}-y_{i}\right)}^{2}}$$

where ${\hat{y}}_{i}$ is the predicted value, $y_{i}$ is the true value and ${\bar{y}}_{i}$ is the average value of the true value, and *m* is the number of predicted samples.

**Figure 10:**
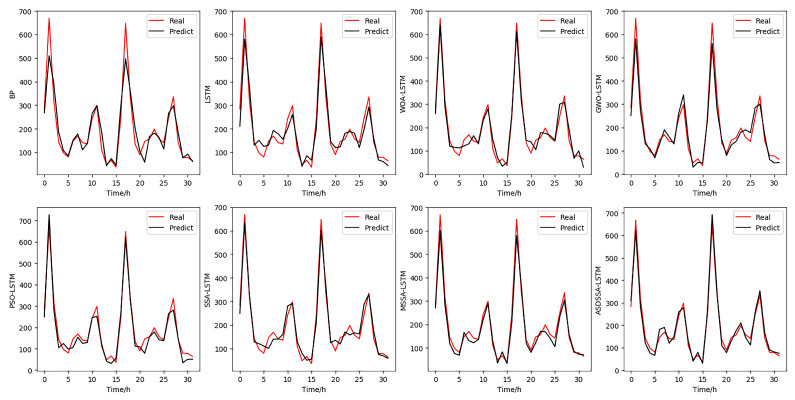
Prediction results of eight models.

Figure 10 shows the experimental results of the eight algorithms on the metro passenger flow dataset, and Table 10 gives the error evaluation results of the eight algorithms. As can be seen in Figure 10, the ASDSSA-LSTM model produced a better fit and better prediction accuracy. As can be seen in Table 10, the basic BP model and the LSTM model have poor prediction results and larger errors. Among the LSTM models improved by the basic swarm optimization algorithm, GWO outperformed the other three algorithms in terms of MAPE metrics, but SSA outperformed the other algorithms in terms of RMSE, MAE and $R^{2}$ metrics, indicating that the SSA-optimized LSTM model is a better fit. The ASDSSA-LSTM model proposed in this paper is superior to other algorithms according to four evaluation indexes. The prediction error measured by MAPE, RMSE and MAE was reduced by at least 20% compared with other optimization algorithms. This shows that the ASDSSA-LSTM model significantly improved the generalization performance and robustness under the same conditions. The superiority of ASDSSA and the effectiveness and feasibility of its application in practice are demonstrated.

## 7. Conclusions

For the shortcomings of insufficient SSA population diversity, slow convergence speed and easy-to-fall-into local optimal solutions, this paper proposes an adaptive sinusoidal-disturbance-strategy sparrow search algorithm. The main research contributions are as follows:

(1) The quality of the initial population of SSA was improved by the cubic chaotic mapping and perturbation compensation factors. The sinusoidal disturbance strategy was introduced to increases the global search capability of the algorithm. The adaptive Cauchy mutation strategy is used to improve the ability to solve the problem of the algorithm easily falling into a local optimal solution in the process of SSA iteration, thereby improving the optimization accuracy and convergence efficiency of the algorithm.
(2) Through the experimental simulation test and the comparison of various algorithms on eight benchmark functions and CEC2017 test functions, it can be found that the improved sparrow algorithm, ASDSSA, greatly improves the search accuracy and convergence speed compared with SSA, and also has obvious advantages compared with other intelligent optimization algorithms, which verifies the improvements brought about by ASDSSA.
(3) ASDSSA was used to optimize the selection of parameters such as learning rate and number of neurons in an LSTM model, and then the ASDSSA-LSTM prediction model was constructed. In the prediction analysis of the Metro passenger flow dataset, the results showed that the improved model has better prediction accuracy, which further verifies the effectiveness and application feasibility of the improved algorithm. In future research, ASDSSA can be used to solve more complex practical problems.

## Figures and Tables

**Table 1:** Parameter settings.

Algorithm	Parameter Setting
WOA	b = 1
GWO	a linearly decreases from 2 to 0, $r_{1}\in$ [0,1], $r_{2}$∈ [0,1]
PSO	$c_{1}$ = 2, $c_{2}$ = 2, $W_{min}$ = 0.2, $W_{max}$ = 0.9
GA	$P_{c}$ = 0.9, $P_{m}$ = 0.1, $K_{g}$ = 1
SSA	N = 30, PD = 0.2, SD = 0.1, ST = 0.8
ASDSSA	N = 30, PD = 0.2, SD = 0.1, w ∈ [1,3], ST = 0.8

**Table 2:** Benchmark test function information.

Test Function	Dim	Scope	Best
$f_{1}\left(x\right)=\sum_{i=1}^{n}X_{i}^{2}$	30	[−100,100]	0
$f_{2}\left(x\right)=\sum_{i=1}^{n}\left|x_{i}\right|+\prod_{i=1}^{n}\left|x_{i}\right|$	30	[−10,10]	0
$f_{3}\left(x\right)=\sum_{i=1}^{n}{\left(\sum_{j=1}^{i}X_{j}\right)}^{2}$	30	[−100,100]	0
$f_{4}\left(x\right)=\max\left(\left|X_{i}\right|,1\leq i\leq n\right)$	30	[−100,100]	0
$f_{5}\left(x\right)=\sum_{i=4}^{n}ix_{i}^{4}+\mathrm{random}\left(0,1\right)$	30	[−1.28,1.28]	0
$f_{6}\left(x\right)=\sum_{i=1}^{n}\left[x_{i}^{2}-10\cos\left(2\pi x_{i}\right)+10\right]$	30	[−5.12,5.12]	0
$f_{7}\left(x\right)=-20\exp\left(-0.2\sqrt{\frac{1}{n}\sum_{i=1}^{n}x_{i}^{2}}\right)-\exp\left(\frac{1}{n}\sum_{i=1}^{n}\cos\left(2\pi x_{i}\right)+20+e\right)$	30	[−32,32]	0
$\begin{array}{c} f_{8}\left(x\right)=\frac{\pi }{n}\left\{10\sin\left(\pi y_{i}\right)+\sum_{i}^{n-1}{\left(y_{i}-1\right)}^{2}\left[1+\sin^{2}\left(\pi y_{i+1}\right)\right]+{\left(y_{n}-1\right)}^{2}\right\}+\\ \sum_{i=1}^{n}u\left(x_{i},10,100,4\right)y_{i}=1+\frac{x_{i}+1}{4} \end{array}$	30	[−50,50]	0

**Table 3:** Comparison of the benchmark test function optimization results.

Function	Result	WOA	GWO	PSO	GA	SSA	ASDSSA
	Best	5.6714 $\times \;10^{-34}$	8.4756 $\times \;10^{-10}$	1.3640 $\times \;10^{-1}$	2.0397 $\times \;10^{2}$	0	0
$f_{1}\left(x\right)$	Mean	1.7242 $\times \;10^{-27}$	4.5327 $\times \;10^{-9}$	2.9247 $\times \;10^{-1}$	3.6529 $\times \;10^{2}$	9.3332 $\times \;10^{-64}$	0
	Std	3.0317 $\times \;10^{-27}$	2.8425 $\times \;10^{-9}$	1.3450 $\times \;10^{-1}$	1.3302 $\times \;10^{2}$	1.9149 $\times \;10^{-64}$	0
	Best	4.6988 $\times \;10^{-32}$	6.7113 $\times \;10^{-6}$	6.3753 $\times \;10^{-1}$	5.0766 $\times \;10^{0}$	0	0
$f_{2}\left(x\right)$	Mean	5.9910 $\times \;10^{-21}$	7.7308 $\times \;10^{-6}$	1.0880 $\times \;10^{0}$	6.3400 $\times \;10^{0}$	5.5920 $\times \;10^{-22}$	0
	Std	1.0619 $\times \;10^{-20}$	5.3187 $\times \;10^{-7}$	4.4150 $\times \;10^{-1}$	8.0122 $\times \;10^{-1}$	9.8272 $\times \;10^{-22}$	0
	Best	6.7168 $\times \;10^{-22}$	3.4165 $\times \;10^{-6}$	5.1550 $\times \;10^{-1}$	3.5946 $\times \;10^{0}$	3.8773 $\times \;10^{-27}$	0
$f_{3}\left(x\right)$	Mean	4.9963 $\times \;10^{-19}$	7.2409 $\times \;10^{-6}$	1.0117 $\times \;10^{0}$	6.5441 $\times \;10^{0}$	3.7489 $\times \;10^{-22}$	0
	Std	9.0402 $\times \;10^{-19}$	3.8888 $\times \;10^{-6}$	4.0392 $\times \;10^{-1}$	1.8641 $\times \;10^{0}$	5.8314 $\times \;10^{-22}$	0
	Best	2.9633 $\times \;10^{-21}$	4.0857 $\times \;10^{-6}$	8.0540 $\times \;10^{-1}$	4.8789 $\times \;10^{0}$	3.2042 $\times \;10^{-37}$	0
$f_{4}\left(x\right)$	Mean	1.9511 $\times \;10^{-19}$	7.4556 $\times \;10^{-6}$	1.3486 $\times \;10^{0}$	6.1209 $\times \;10^{0}$	3.7929 $\times \;10^{-23}$	0
	Std	2.2805 $\times \;10^{-19}$	2.0061 $\times \;10^{-6}$	4.1610 $\times \;10^{-1}$	6.5100 $\times \;10^{-1}$	7.3201 $\times \;10^{-23}$	0
	Best	4.0528 $\times \;10^{-21}$	4.1442 $\times \;10^{-6}$	6.3050 $\times \;10^{-1}$	5.9261 $\times \;10^{0}$	5.4651 $\times \;10^{-31}$	6.3410 $\times \;10^{-80}$
$f_{5}\left(x\right)$	Mean	8.1200 $\times \;10^{-20}$	8.1688 $\times \;10^{-6}$	1.0497 $\times \;10^{0}$	7.0711 $\times \;10^{0}$	3.4242 $\times \;10^{-21}$	1.5806 $\times \;10^{-31}$
	Std	1.0386 $\times \;10^{-19}$	3.2248 $\times \;10^{-6}$	2.9401 $\times \;10^{-1}$	9.9521 $\times \;10^{-1}$	6.8234 $\times \;10^{-21}$	3.1613 $\times \;10^{-31}$
	Best	5.4927 $\times \;10^{-22}$	2.4677 $\times \;10^{-6}$	7.1291 $\times \;10^{-1}$	5.5451 $\times \;10^{0}$	0	0
$f_{6}\left(x\right)$	Mean	1.8230 $\times \;10^{-21}$	5.7594 $\times \;10^{-6}$	1.4673 $\times \;10^{0}$	6.5011 $\times \;10^{0}$	9.7408 $\times \;10^{-26}$	0
	Std	2.2398 $\times \;10^{-21}$	2.4299 $\times \;10^{-6}$	4.3940 $\times \;10^{-1}$	7.9890 $\times \;10^{-1}$	1.2845 $\times \;10^{-25}$	0
	Best	5.6347 $\times \;10^{-23}$	3.8513 $\times \;10^{-6}$	7.1592 $\times \;10^{-1}$	4.8912 $\times \;10^{0}$	5.9900 $\times \;10^{-39}$	0
$f_{7}\left(x\right)$	Mean	1.2392 $\times \;10^{-19}$	6.1325 $\times \;10^{-6}$	1.3973 $\times \;10^{0}$	6.2328 $\times \;10^{0}$	2.9862 $\times \;10^{-23}$	3.6154 $\times \;10^{-39}$
	Std	1.3452 $\times \;10^{-19}$	1.6556 $\times \;10^{-6}$	5.2793 $\times \;10^{-1}$	9.0850 $\times \;10^{-1}$	5.9409 $\times \;10^{-23}$	7.2308 $\times \;10^{-39}$
	Best	1.0030 $\times \;10^{-21}$	3.0763 $\times \;10^{-6}$	7.3255 $\times \;10^{-1}$	4.3292 $\times \;10^{0}$	1.6478 $\times \;10^{-30}$	0
$f_{8}\left(x\right)$	Mean	6.2581 $\times \;10^{-20}$	7.0388 $\times \;10^{-6}$	1.1587 $\times \;10^{0}$	6.0629 $\times \;10^{0}$	2.0488 $\times \;10^{-22}$	1.1446 $\times \;10^{-38}$
	Std	1.2154 $\times \;10^{-19}$	2.2969 $\times \;10^{-6}$	3.5971 $\times \;10^{-1}$	1.5728 $\times \;10^{0}$	2.2609 $\times \;10^{-22}$	2.2892 $\times \;10^{-38}$

**Table 4:** The performances of the HOS+ algorithm and the ASDSSA using different numbers of dimensions.

			HOS+			ASDSSA	
**Benchmark Functions**	**D**	**Best**	**Mean**	**Std**	**Best**	**Mean**	**Std**
	30	9.95 $\times \;10^{-137}$	2.63 $\times \;10^{-51}$	4.29 $\times \;10^{-51}$	0	0	0
Sphere	60	7.79 $\times \;10^{-96}$	6.39 $\times \;10^{-39}$	6.19 $\times \;10^{-39}$	0	0	0
	90	1.70 $\times \;10^{-81}$	2.19 $\times \;10^{-32}$	3.59 $\times \;10^{-32}$	0	3.87 $\times \;10^{-109}$	8.65 $\times \;10^{-109}$
	30	1.19 $\times \;10^{-95}$	1.98 $\times \;10^{-27}$	3.19 $\times \;10^{-27}$	0	0	0
Schwefel 2.22	60	3.21 $\times \;10^{-72}$	3.88 $\times \;10^{-22}$	6.39 $\times \;10^{-22}$	0	1.19 $\times \;10^{-97}$	2.66 $\times \;10^{-97}$
	90	2.49 $\times \;10^{-59}$	5.72 $\times \;10^{-18}$	6.91 $\times \;10^{-18}$	0	3.20 $\times \;10^{-125}$	6.40 $\times \;10^{-125}$
	30	8.88 $\times \;10^{-16}$	3.57 $\times \;10^{-15}$	4.69 $\times \;10^{-15}$	8.88 $\times \;10^{-16}$	8.88 $\times \;10^{-16}$	0
Ackley	60	8.88 $\times \;10^{-16}$	2.53 $\times \;10^{-14}$	2.79 $\times \;10^{-14}$	8.88 $\times \;10^{-16}$	8.88 $\times \;10^{-16}$	0
	90	4.44 $\times \;10^{-15}$	4.29 $\times \;10^{-13}$	6.69 $\times \;10^{-13}$	8.88 $\times \;10^{-16}$	8.88 $\times \;10^{-16}$	0
	30	0	0	0	0	0	0
Griewank	60	0	0	0	0	0	0
	90	0	0	0	0	0	0
Hyper	30	1.55 $\times \;10^{-139}$	2.19 $\times \;10^{-48}$	1.77 $\times \;10^{-48}$	0	0	0
Ellipsoid	60	4.35 $\times \;10^{-113}$	3.39 $\times \;10^{-36}$	3.59 $\times \;10^{-36}$	0	0	0
	90	4.89 $\times \;10^{-79}$	2.99 $\times \;10^{-29}$	3.96 $\times \;10^{-29}$	0	1.22 $\times \;10^{-164}$	2.82 $\times \;10^{-164}$
Rotated	30	3.39 $\times \;10^{-134}$	2.69 $\times \;10^{-47}$	2.03 $\times \;10^{-47}$	0	0	0
Hyper	60	5.25 $\times \;10^{-109}$	1.75 $\times \;10^{-34}$	2.09 $\times \;10^{-34}$	0	0	0
Ellipsoid	90	1.05 $\times \;10^{-75}$	1.35 $\times \;10^{-28}$	1.79 $\times \;10^{-28}$	0	8.09 $\times \;10^{-226}$	9.93 $\times \;10^{-226}$

**Table 5:** CEC2017 test results.

Function	Index	WOA	GWO	PSO	SSA	MSSA	ASDSSA
	Mean	4.73 $\times \;10^{5}$	6.95 $\times \;10^{6}$	4.97 $\times \;10^{3}$	3.95 $\times \;10^{3}$	3.29 $\times \;10^{3}$	1.32 $\times \;10^{2}$
F1	Std	3.98 $\times \;10^{5}$	5.53 $\times \;10^{6}$	2.35 $\times \;10^{3}$	3.34 $\times \;10^{2}$	3.01 $\times \;10^{2}$	8.59 $\times \;10^{0}$
	Mean	2.99 $\times \;10^{4}$	1.78 $\times \;10^{4}$	3.13 $\times \;10^{2}$	7.79 $\times \;10^{2}$	5.59 $\times \;10^{2}$	3.04 $\times \;10^{2}$
F3	Std	1.39 $\times \;10^{4}$	7.85 $\times \;10^{3}$	7.85 $\times \;10^{0}$	4.23 $\times \;10^{1}$	1.77 $\times \;10^{1}$	4.26 $\times \;10^{0}$
	Mean	6.34 $\times \;10^{2}$	9.21 $\times \;10^{2}$	6.83 $\times \;10^{2}$	6.27 $\times \;10^{2}$	4.98 $\times \;10^{2}$	4.87 $\times \;10^{2}$
F4	Std	4.45 $\times \;10^{1}$	5.61 $\times \;10^{1}$	3.79 $\times \;10^{1}$	2.05 $\times \;10^{1}$	2.97 $\times \;10^{1}$	1.78 $\times \;10^{1}$
	Mean	7.74 $\times \;10^{2}$	7.21 $\times \;10^{2}$	7.56 $\times \;10^{2}$	6.89 $\times \;10^{2}$	7.59 $\times \;10^{2}$	5.94 $\times \;10^{2}$
F5	Std	3.56 $\times \;10^{1}$	1.92 $\times \;10^{1}$	3.62 $\times \;10^{1}$	4.17 $\times \;10^{1}$	3.98 $\times \;10^{1}$	3.72 $\times \;10^{1}$
	Mean	6.76 $\times \;10^{2}$	6.70 $\times \;10^{2}$	6.52 $\times \;10^{2}$	6.19 $\times \;10^{2}$	6.49 $\times \;10^{2}$	6.28 $\times \;10^{2}$
F6	Std	1.25 $\times \;10^{1}$	5.29 $\times \;10^{0}$	9.34 $\times \;10^{0}$	1.01 $\times \;10^{1}$	5.76 $\times \;10^{0}$	2.61 $\times \;10^{0}$
	Mean	1.25 $\times \;10^{3}$	1.27 $\times \;10^{3}$	1.16 $\times \;10^{3}$	1.17 $\times \;10^{3}$	9.87 $\times \;10^{2}$	8.85 $\times \;10^{2}$
F7	Std	4.33 $\times \;10^{1}$	6.81 $\times \;10^{1}$	2.55 $\times \;10^{1}$	3.47 $\times \;10^{1}$	2.48 $\times \;10^{1}$	2.43 $\times \;10^{1}$
	Mean	1.01 $\times \;10^{3}$	9.92 $\times \;10^{2}$	9.96 $\times \;10^{2}$	9.87 $\times \;10^{2}$	9.32 $\times \;10^{2}$	9.20 $\times \;10^{2}$
F8	Std	4.14 $\times \;10^{1}$	1.67 $\times \;10^{1}$	3.43 $\times \;10^{1}$	3.83 $\times \;10^{1}$	1.72 $\times \;10^{1}$	2.23 $\times \;10^{1}$
	Mean	2.33 $\times \;10^{3}$	5.53 $\times \;10^{3}$	3.10 $\times \;10^{3}$	4.72 $\times \;10^{3}$	9.82 $\times \;10^{2}$	1.11 $\times \;10^{3}$
F9	Std	5.92 $\times \;10^{1}$	6.77 $\times \;10^{1}$	1.77 $\times \;10^{2}$	1.41 $\times \;10^{2}$	2.63 $\times \;10^{1}$	1.93 $\times \;10^{1}$
	Mean	6.40 $\times \;10^{3}$	7.76 $\times \;10^{3}$	8.76 $\times \;10^{3}$	4.83 $\times \;10^{3}$	6.73 $\times \;10^{3}$	4.19 $\times \;10^{3}$
F10	Std	6.32 $\times \;10^{2}$	6.45 $\times \;10^{2}$	7.91 $\times \;10^{2}$	8.14 $\times \;10^{2}$	7.85 $\times \;10^{2}$	5.37 $\times \;10^{2}$
	Mean	1.39 $\times \;10^{3}$	1.53 $\times \;10^{3}$	1.28 $\times \;10^{3}$	1.18 $\times \;10^{3}$	1.22 $\times \;10^{3}$	1.12 $\times \;10^{3}$
F11	Std	1.71 $\times \;10^{1}$	2.39 $\times \;10^{1}$	7.91 $\times \;10^{1}$	5.47 $\times \;10^{1}$	1.03 $\times \;10^{1}$	5.39 $\times \;10^{1}$
	Mean	1.34 $\times \;10^{8}$	2.97 $\times \;10^{8}$	4.88 $\times \;10^{9}$	7.76 $\times \;10^{4}$	4.63 $\times \;10^{7}$	6.72 $\times \;10^{5}$
F12	Std	8.47 $\times \;10^{7}$	8.28 $\times \;10^{7}$	2.04 $\times \;10^{9}$	2.12 $\times \;10^{4}$	3.48 $\times \;10^{7}$	1.65 $\times \;10^{5}$
	Mean	5.92 $\times \;10^{5}$	1.39 $\times \;10^{8}$	4.95 $\times \;10^{8}$	4.18 $\times \;10^{5}$	1.99 $\times \;10^{4}$	3.02 $\times \;10^{3}$
F13	Std	5.15 $\times \;10^{5}$	4.60 $\times \;10^{7}$	6.74 $\times \;10^{8}$	1.31 $\times \;10^{6}$	7.60 $\times \;10^{4}$	2.49 $\times \;10^{3}$
	Mean	1.83 $\times \;10^{5}$	3.66 $\times \;10^{5}$	1.18 $\times \;10^{4}$	1.95 $\times \;10^{3}$	5.61 $\times \;10^{3}$	4.01 $\times \;10^{3}$
F14	Std	1.85 $\times \;10^{5}$	6.03 $\times \;10^{5}$	2.36 $\times \;10^{4}$	2.49 $\times \;10^{3}$	3.69 $\times \;10^{4}$	3.47 $\times \;10^{3}$
	Mean	3.20 $\times \;10^{5}$	2.72 $\times \;10^{5}$	1.20 $\times \;10^{4}$	5.57 $\times \;10^{3}$	2.52 $\times \;10^{3}$	5.76 $\times \;10^{3}$
F15	Std	4.48 $\times \;10^{5}$	1.83 $\times \;10^{4}$	8.93 $\times \;10^{3}$	3.59 $\times \;10^{2}$	1.10 $\times \;10^{3}$	4.29 $\times \;10^{3}$
	Mean	3.74 $\times \;10^{3}$	3.06 $\times \;10^{3}$	3.72 $\times \;10^{3}$	2.26 $\times \;10^{3}$	4.32 $\times \;10^{3}$	2.92 $\times \;10^{3}$
F16	Std	2.67 $\times \;10^{2}$	2.67 $\times \;10^{2}$	4.10 $\times \;10^{2}$	2.93 $\times \;10^{2}$	8.84 $\times \;10^{2}$	3.54 $\times \;10^{2}$
	Mean	2.62 $\times \;10^{3}$	2.18 $\times \;10^{3}$	2.48 $\times \;10^{3}$	2.93 $\times \;10^{3}$	2.19 $\times \;10^{3}$	1.83 $\times \;10^{3}$
F17	Std	2.91 $\times \;10^{1}$	1.49 $\times \;10^{1}$	2.72 $\times \;10^{1}$	3.86 $\times \;10^{1}$	2.69 $\times \;10^{1}$	2.66 $\times \;10^{1}$
	Mean	6.07 $\times \;10^{6}$	2.78 $\times \;10^{6}$	3.41 $\times \;10^{6}$	4.72 $\times \;10^{6}$	4.09 $\times \;10^{5}$	8.69 $\times \;10^{4}$
F18	Std	6.80 $\times \;10^{6}$	3.57 $\times \;10^{6}$	7.36 $\times \;10^{6}$	6.59 $\times \;10^{6}$	1.09 $\times \;10^{5}$	9.66 $\times \;10^{4}$
	Mean	1.45 $\times \;10^{5}$	2.88 $\times \;10^{5}$	1.06 $\times \;10^{4}$	2.24 $\times \;10^{3}$	2.09 $\times \;10^{3}$	2.00 $\times \;10^{3}$
F19	Std	5.54 $\times \;10^{4}$	2.07 $\times \;10^{5}$	8.67 $\times \;10^{3}$	4.28 $\times \;10^{2}$	8.73 $\times \;10^{2}$	1.16 $\times \;10^{2}$
	Mean	2.99 $\times \;10^{3}$	5.22 $\times \;10^{3}$	5.71 $\times \;10^{3}$	2.14 $\times \;10^{3}$	2.12 $\times \;10^{3}$	2.06 $\times \;10^{3}$
F20	Std	7.89 $\times \;10^{1}$	2.35 $\times \;10^{2}$	2.32 $\times \;10^{2}$	8.31 $\times \;10^{1}$	8.34 $\times \;10^{1}$	5.34 $\times \;10^{1}$
	Mean	2.58 $\times \;10^{3}$	2.50 $\times \;10^{3}$	2.57 $\times \;10^{3}$	2.38 $\times \;10^{3}$	2.59 $\times \;10^{3}$	2.31 $\times \;10^{3}$
F21	Std	4.21 $\times \;10^{1}$	2.12 $\times \;10^{1}$	3.64 $\times \;10^{1}$	4.16 $\times \;10^{1}$	5.14 $\times \;10^{1}$	6.67 $\times \;10^{1}$
	Mean	5.99 $\times \;10^{3}$	7.11 $\times \;10^{3}$	3.97 $\times \;10^{3}$	2.35 $\times \;10^{3}$	2.42 $\times \;10^{3}$	2.30 $\times \;10^{3}$
F22	Std	1.94 $\times \;10^{2}$	2.72 $\times \;10^{2}$	2.13 $\times \;10^{2}$	2.35 $\times \;10^{2}$	9.38 $\times \;10^{1}$	1.41 $\times \;10^{1}$
	Mean	3.08 $\times \;10^{3}$	2.90 $\times \;10^{3}$	3.51 $\times \;10^{3}$	2.75 $\times \;10^{3}$	3.35 $\times \;10^{3}$	2.65 $\times \;10^{3}$
F23	Std	8.93 $\times \;10^{2}$	2.58 $\times \;10^{1}$	1.46 $\times \;10^{2}$	6.87 $\times \;10^{1}$	1.39 $\times \;10^{2}$	2.65 $\times \;10^{1}$
	Mean	3.22 $\times \;10^{3}$	3.07 $\times \;10^{3}$	3.85 $\times \;10^{3}$	3.17 $\times \;10^{3}$	2.90 $\times \;10^{3}$	2.76 $\times \;10^{3}$
F24	Std	9.23 $\times \;10^{2}$	2.38 $\times \;10^{2}$	1.63 $\times \;10^{2}$	1.37 $\times \;10^{2}$	6.51 $\times \;10^{2}$	1.17 $\times \;10^{2}$
	Mean	3.62 $\times \;10^{3}$	3.04 $\times \;10^{3}$	3.05 $\times \;10^{3}$	2.91 $\times \;10^{3}$	2.88 $\times \;10^{3}$	2.92 $\times \;10^{3}$
F25	Std	2.19 $\times \;10^{1}$	4.57 $\times \;10^{1}$	4.15 $\times \;10^{1}$	2.22 $\times \;10^{1}$	1.63 $\times \;10^{1}$	5.37 $\times \;10^{1}$
	Mean	8.76 $\times \;10^{3}$	5.91 $\times \;10^{3}$	6.33 $\times \;10^{3}$	6.04 $\times \;10^{3}$	2.81 $\times \;10^{3}$	3.36 $\times \;10^{3}$
F26	Std	7.02 $\times \;10^{2}$	5.56 $\times \;10^{2}$	9.90 $\times \;10^{2}$	1.08 $\times \;10^{3}$	1.52 $\times \;10^{3}$	3.70 $\times \;10^{2}$
	Mean	3.39 $\times \;10^{3}$	4.39 $\times \;10^{3}$	3.29 $\times \;10^{3}$	3.30 $\times \;10^{3}$	3.22 $\times \;10^{3}$	3.15 $\times \;10^{3}$
F27	Std	9.96 $\times \;10^{1}$	3.26 $\times \;10^{2}$	2.29 $\times \;10^{1}$	3.36 $\times \;10^{1}$	2.07 $\times \;10^{1}$	5.29 $\times \;10^{1}$
	Mean	5.25 $\times \;10^{3}$	3.51 $\times \;10^{3}$	3.47 $\times \;10^{3}$	3.21 $\times \;10^{3}$	3.89 $\times \;10^{3}$	3.27 $\times \;10^{3}$
F28	Std	5.50 $\times \;10^{2}$	7.66 $\times \;10^{1}$	7.39 $\times \;10^{1}$	2.31 $\times \;10^{1}$	2.76 $\times \;10^{2}$	1.36 $\times \;10^{2}$
	Mean	5.11 $\times \;10^{3}$	5.39 $\times \;10^{3}$	4.28 $\times \;10^{3}$	4.43 $\times \;10^{3}$	3.74 $\times \;10^{3}$	3.33 $\times \;10^{3}$
F29	Std	4.12 $\times \;10^{2}$	5.08 $\times \;10^{2}$	1.97 $\times \;10^{2}$	6.41 $\times \;10^{2}$	1.72 $\times \;10^{2}$	9.02 $\times \;10^{1}$
	Mean	1.41 $\times \;10^{6}$	2.82 $\times \;10^{6}$	4.41 $\times \;10^{4}$	7.21 $\times \;10^{3}$	6.92 $\times \;10^{3}$	4.08 $\times \;10^{3}$
F30	Std	2.76 $\times \;10^{6}$	9.13 $\times \;10^{6}$	2.76 $\times \;10^{4}$	1.24 $\times \;10^{3}$	1.10 $\times \;10^{3}$	1.24 $\times \;10^{3}$

**Table 6:** The test results of ASDSSA with Dim = 10, Dim = 50 and Dim = 100 on CEC2017.

		Dim = 10			Dim = 50			Dim = 100	
**Function**	**Best**	**Mean**	**Std**	**Best**	**Mean**	**Std**	**Best**	**Mean**	**Std**
F1	1.00 $\times \;10^{2}$	1.00 $\times \;10^{2}$	0	2.03 $\times \;10^{2}$	1.34 $\times \;10^{3}$	1.33 $\times \;10^{3}$	1.08 $\times \;10^{2}$	4.73 $\times \;10^{4}$	2.31 $\times \;10^{4}$
F3	3.00 $\times \;10^{2}$	3.00 $\times \;10^{2}$	0	4.37 $\times \;10^{2}$	5.58 $\times \;10^{0}$	2.35 $\times \;10^{2}$	3.17 $\times \;10^{4}$	5.32 $\times \;10^{4}$	1.27 $\times \;10^{4}$
F4	4.00 $\times \;10^{2}$	4.00 $\times \;10^{2}$	0	4.97 $\times \;10^{2}$	5.64 $\times \;10^{2}$	5.71 $\times \;10^{1}$	1.17 $\times \;10^{3}$	1.40 $\times \;10^{3}$	1.76 $\times \;10^{2}$
F5	5.00 $\times \;10^{2}$	5.14 $\times \;10^{2}$	8.78 $\times \;10^{0}$	5.09 $\times \;10^{2}$	8.64 $\times \;10^{2}$	2.16 $\times \;10^{1}$	1.27 $\times \;10^{3}$	1.38 $\times \;10^{3}$	3.69 $\times \;10^{1}$
F6	6.00 $\times \;10^{2}$	6.00 $\times \;10^{2}$	3.04 $\times \;10^{0}$	6.39 $\times \;10^{2}$	6.60 $\times \;10^{2}$	6.59 $\times \;10^{0}$	6.63 $\times \;10^{2}$	6.67 $\times \;10^{2}$	2.25 $\times \;10^{0}$
F7	7.20 $\times \;10^{2}$	7.27 $\times \;10^{2}$	3.04 $\times \;10^{1}$	8.06 $\times \;10^{2}$	1.02 $\times \;10^{3}$	8.67 $\times \;10^{1}$	2.69 $\times \;10^{3}$	3.23 $\times \;10^{3}$	1.43 $\times \;10^{2}$
F8	8.00 $\times \;10^{2}$	8.23 $\times \;10^{2}$	7.24 $\times \;10^{0}$	1.03 $\times \;10^{3}$	1.18 $\times \;10^{3}$	4.70 $\times \;10^{1}$	1.70 $\times \;10^{3}$	1.86 $\times \;10^{3}$	5.20 $\times \;10^{1}$
F9	9.00 $\times \;10^{2}$	1.19 $\times \;10^{3}$	3.83 $\times \;10^{2}$	9.09 $\times \;10^{2}$	9.53 $\times \;10^{2}$	3.03 $\times \;10^{1}$	2.54 $\times \;10^{3}$	2.71 $\times \;10^{3}$	1.37 $\times \;10^{3}$
F10	1.12 $\times \;10^{3}$	1.34 $\times \;10^{3}$	1.66 $\times \;10^{3}$	5.98 $\times \;10^{3}$	8.61 $\times \;10^{3}$	1.05 $\times \;10^{3}$	1.37 $\times \;10^{4}$	1.81 $\times \;10^{4}$	1.65 $\times \;10^{3}$
F11	1.10 $\times \;10^{3}$	1.10 $\times \;10^{3}$	0	1.11 $\times \;10^{3}$	1.12 $\times \;10^{3}$	1.09 $\times \;10^{1}$	4.58 $\times \;10^{4}$	7.49 $\times \;10^{4}$	1.51 $\times \;10^{4}$
F12	1.64 $\times \;10^{3}$	1.67 $\times \;10^{3}$	4.32 $\times \;10^{1}$	5.63 $\times \;10^{3}$	1.00 $\times \;10^{4}$	8.81 $\times \;10^{4}$	1.57 $\times \;10^{4}$	7.49 $\times \;10^{4}$	1.51 $\times \;10^{4}$
F13	1.30 $\times \;10^{3}$	1.31 $\times \;10^{3}$	2.57 $\times \;10^{0}$	1.31 $\times \;10^{3}$	8.25 $\times \;10^{3}$	2.40 $\times \;10^{3}$	9.81 $\times \;10^{3}$	2.88 $\times \;10^{4}$	1.84 $\times \;10^{4}$
F14	1.41 $\times \;10^{3}$	1.41 $\times \;10^{3}$	3.18 $\times \;10^{0}$	1.46 $\times \;10^{3}$	1.55 $\times \;10^{3}$	9.23 $\times \;10^{1}$	9.34 $\times \;10^{3}$	1.03 $\times \;10^{4}$	2.77 $\times \;10^{3}$
F15	1.51 $\times \;10^{3}$	1.58 $\times \;10^{3}$	4.08 $\times \;10^{1}$	1.60 $\times \;10^{3}$	1.68 $\times \;10^{3}$	9.67 $\times \;10^{1}$	1.43 $\times \;10^{4}$	3.28 $\times \;10^{4}$	1.52 $\times \;10^{4}$
F16	1.60 $\times \;10^{3}$	1.61 $\times \;10^{3}$	1.42 $\times \;10^{1}$	1.99 $\times \;10^{3}$	2.02 $\times \;10^{3}$	3.91 $\times \;10^{1}$	3.35 $\times \;10^{3}$	3.85 $\times \;10^{3}$	6.87 $\times \;10^{2}$
F17	1.70 $\times \;10^{3}$	1.71 $\times \;10^{3}$	1.87 $\times \;10^{1}$	2.19 $\times \;10^{3}$	2.43 $\times \;10^{3}$	3.21 $\times \;10^{2}$	4.78 $\times \;10^{3}$	5.76 $\times \;10^{3}$	5.92 $\times \;10^{2}$
F18	1.85 $\times \;10^{3}$	1.88 $\times \;10^{3}$	2.38 $\times \;10^{1}$	2.90 $\times \;10^{3}$	3.39 $\times \;10^{3}$	2.61 $\times \;10^{2}$	5.87 $\times \;10^{4}$	6.00 $\times \;10^{4}$	3.63 $\times \;10^{3}$
F19	1.90 $\times \;10^{3}$	1.91 $\times \;10^{3}$	1.48 $\times \;10^{1}$	2.03 $\times \;10^{3}$	1.03 $\times \;10^{4}$	8.63 $\times \;10^{3}$	2.56 $\times \;10^{3}$	9.53 $\times \;10^{3}$	1.77 $\times \;10^{4}$
F20	2.20 $\times \;10^{3}$	2.01 $\times \;10^{3}$	1.40 $\times \;10^{1}$	2.80 $\times \;10^{3}$	3.23 $\times \;10^{3}$	2.92 $\times \;10^{2}$	4.14 $\times \;10^{3}$	6.21 $\times \;10^{3}$	6.28 $\times \;10^{2}$
F21	2.10 $\times \;10^{3}$	2.28 $\times \;10^{3}$	6.49 $\times \;10^{1}$	2.45 $\times \;10^{3}$	2.65 $\times \;10^{3}$	5.99 $\times \;10^{1}$	2.14 $\times \;10^{3}$	2.46 $\times \;10^{3}$	1.87 $\times \;10^{2}$
F22	2.23 $\times \;10^{3}$	2.30 $\times \;10^{3}$	1.29 $\times \;10^{1}$	2.23 $\times \;10^{3}$	4.03 $\times \;10^{3}$	1.45 $\times \;10^{3}$	1.74 $\times \;10^{4}$	2.17 $\times \;10^{4}$	1.92 $\times \;10^{3}$
F23	2.60 $\times \;10^{3}$	2.62 $\times \;10^{3}$	1.08 $\times \;10^{1}$	2.98 $\times \;10^{3}$	3.20 $\times \;10^{3}$	1.08 $\times \;10^{2}$	3.68 $\times \;10^{3}$	3.94 $\times \;10^{3}$	1.81 $\times \;10^{2}$
F24	2.50 $\times \;10^{3}$	2.74 $\times \;10^{3}$	6.33 $\times \;10^{1}$	3.01 $\times \;10^{3}$	3.03 $\times \;10^{3}$	1.07 $\times \;10^{2}$	4.25 $\times \;10^{3}$	4.77 $\times \;10^{3}$	2.57 $\times \;10^{2}$
F25	2.60 $\times \;10^{3}$	2.91 $\times \;10^{3}$	5.01 $\times \;10^{1}$	2.96 $\times \;10^{3}$	3.04 $\times \;10^{3}$	3.56 $\times \;10^{1}$	2.96 $\times \;10^{3}$	2.97 $\times \;10^{3}$	1.29 $\times \;10^{1}$
F26	2.60 $\times \;10^{3}$	2.98 $\times \;10^{3}$	1.74 $\times \;10^{1}$	3.09 $\times \;10^{3}$	7.28 $\times \;10^{3}$	3.32 $\times \;10^{3}$	3.52 $\times \;10^{3}$	4.39 $\times \;10^{3}$	2.93 $\times \;10^{2}$
F27	3.08 $\times \;10^{3}$	3.10 $\times \;10^{3}$	1.80 $\times \;10^{1}$	3.23 $\times \;10^{3}$	3.28 $\times \;10^{3}$	1.43 $\times \;10^{2}$	3.34 $\times \;10^{3}$	3.48 $\times \;10^{3}$	6.24 $\times \;10^{1}$
F28	3.10 $\times \;10^{3}$	3.15 $\times \;10^{3}$	1.53 $\times \;10^{1}$	3.27 $\times \;10^{3}$	3.35 $\times \;10^{3}$	4.98 $\times \;10^{1}$	3.94 $\times \;10^{3}$	4.22 $\times \;10^{3}$	1.90 $\times \;10^{2}$
F29	3.14 $\times \;10^{3}$	3.15 $\times \;10^{3}$	6.46 $\times \;10^{0}$	3.88 $\times \;10^{3}$	3.23 $\times \;10^{3}$	4.21 $\times \;10^{1}$	3.85 $\times \;10^{3}$	3.99 $\times \;10^{3}$	1.76 $\times \;10^{2}$
F30	4.27 $\times \;10^{3}$	5.71 $\times \;10^{3}$	1.44 $\times \;10^{3}$	3.41 $\times \;10^{5}$	6.59 $\times \;10^{5}$	5.93 $\times \;10^{4}$	1.34 $\times \;10^{4}$	2.62 $\times \;10^{4}$	2.37 $\times \;10^{4}$

**Table 7:** Results of ASDSSA vs. GSK.

ASDSSA vs. GSK	+	≈	−
Dim = 10	13	9	7
Dim = 30	14	5	10
Dim = 50	13	5	11
Dim = 100	9	6	14

**Table 8:** Results of ASDSSA vs. LSHADE.

ASDSSA vs. LSHADE	+	≈	−
Dim=10	12	8	9
Dim=30	10	11	8
Dim=50	14	7	8
Dim=100	17	3	9

**Table 9:** Wilcoxon rank-sum test results.

Function	ASDSSA-WOA	ASDSSA-GWO	ASDSSA-PSO	ASDSSA-SSA	ASDSSA-MSSA
F1	3.20 $\times \;10^{-18}$	3.20 $\times \;10^{-18}$	3.26 $\times \;10^{-18}$	1.77 $\times \;10^{-10}$	3.22 $\times \;10^{-18}$
F3	3.19 $\times \;10^{-18}$	1.09 $\times \;10^{-5}$	8.15 $\times \;10^{-15}$	4.11 $\times \;10^{-2}$	4.18 $\times \;10^{-1}$
F4	4.58 $\times \;10^{-17}$	1.63 $\times \;10^{-17}$	3.24 $\times \;10^{-18}$	2.14 $\times \;10^{-11}$	3.18 $\times \;10^{-18}$
F5	1.95 $\times \;10^{-7}$	8.62 $\times \;10^{-7}$	2.72 $\times \;10^{-2}$	2.13 $\times \;10^{-2}$	3.67 $\times \;10^{-2}$
F6	3.20 $\times \;10^{-18}$	6.53 $\times \;10^{-18}$	2.79 $\times \;10^{-6}$	1.18 $\times \;10^{-11}$	4.18 $\times \;10^{-5}$
F7	2.84 $\times \;10^{-8}$	3.19 $\times \;10^{-18}$	3.25 $\times \;10^{-18}$	1.87 $\times \;10^{-4}$	2.87 $\times \;10^{-1}$
F8	2.26 $\times \;10^{-8}$	2.36 $\times \;10^{-6}$	3.92 $\times \;10^{-2}$	2.21 $\times \;10^{-1}$	8.76 $\times \;10^{-4}$
F9	9.06 $\times \;10^{-16}$	4.63 $\times \;10^{-18}$	1.20 $\times \;10^{-8}$	1.39 $\times \;10^{-8}$	2.61 $\times \;10^{-4}$
F10	1.02 $\times \;10^{-5}$	2.01 $\times \;10^{-16}$	3.67 $\times \;10^{-18}$	3.33 $\times \;10^{-4}$	5.38 $\times \;10^{-4}$
F11	3.24 $\times \;10^{-18}$	5.19 $\times \;10^{-18}$	3.27 $\times \;10^{-18}$	9.51 $\times \;10^{-5}$	3.24 $\times \;10^{-18}$
F12	3.71 $\times \;10^{-13}$	3.17 $\times \;10^{-18}$	3.23 $\times \;10^{-18}$	1.72 $\times \;10^{-17}$	1.52 $\times \;10^{-15}$
F13	2.06 $\times \;10^{-13}$	3.21 $\times \;10^{-18}$	1.17 $\times \;10^{-12}$	6.78 $\times \;10^{-16}$	8.62 $\times \;10^{-1}$
F14	6.08 $\times \;10^{-8}$	4.33 $\times \;10^{-18}$	1.58 $\times \;10^{-5}$	3.52 $\times \;10^{-9}$	7.27 $\times \;10^{-9}$
F15	2.01 $\times \;10^{-15}$	3.21 $\times \;10^{-18}$	3.88 $\times \;10^{-12}$	5.56 $\times \;10^{-13}$	7.28 $\times \;10^{-2}$
F16	5.82 $\times \;10^{-9}$	1.08 $\times \;10^{-1}$	3.59 $\times \;10^{-1}$	3.31 $\times \;10^{-2}$	1.25 $\times \;10^{-14}$
F17	8.55 $\times \;10^{-13}$	3.55 $\times \;10^{-1}$	2.55 $\times \;10^{-9}$	7.28 $\times \;10^{-8}$	2.95 $\times \;10^{-2}$
F18	1.13 $\times \;10^{-10}$	4.17 $\times \;10^{-5}$	4.86 $\times \;10^{-5}$	2.37 $\times \;10^{-4}$	8.51 $\times \;10^{-1}$
F19	2.77 $\times \;10^{-9}$	4.89 $\times \;10^{-9}$	4.43 $\times \;10^{-18}$	5.42 $\times \;10^{-5}$	4.68 $\times \;10^{-2}$
F20	1.79 $\times \;10^{-8}$	1.05 $\times \;10^{-9}$	9.07 $\times \;10^{-10}$	8.30 $\times \;10^{-2}$	1.94 $\times \;10^{-6}$
F21	4.37 $\times \;10^{-17}$	3.89 $\times \;10^{-11}$	2.36 $\times \;10^{-17}$	8.63 $\times \;10^{-17}$	1.17 $\times \;10^{-15}$
F22	7.96 $\times \;10^{-11}$	1.43 $\times \;10^{-16}$	6.19 $\times \;10^{-13}$	3.65 $\times \;10^{-12}$	1.29 $\times \;10^{-6}$
F23	1.68 $\times \;10^{-16}$	3.24 $\times \;10^{-18}$	4.52 $\times \;10^{-18}$	3.10 $\times \;10^{-3}$	4.26 $\times \;10^{-18}$
F24	1.16 $\times \;10^{-14}$	2.74 $\times \;10^{-9}$	3.24 $\times \;10^{-18}$	4.25 $\times \;10^{-18}$	3.24 $\times \;10^{-17}$
F25	3.17 $\times \;10^{-18}$	3.17 $\times \;10^{-18}$	3.24 $\times \;10^{-18}$	5.43 $\times \;10^{-17}$	3.18 $\times \;10^{-18}$
F26	1.78 $\times \;10^{-11}$	7.48 $\times \;10^{-16}$	1.48 $\times \;10^{-17}$	5.38 $\times \;10^{-2}$	5.74 $\times \;10^{-17}$
F27	1.93 $\times \;10^{-17}$	1.53 $\times \;10^{-12}$	3.24 $\times \;10^{-18}$	3.17 $\times \;10^{-18}$	3.17 $\times \;10^{-18}$
F28	4.32 $\times \;10^{-18}$	3.20 $\times \;10^{-18}$	3.25 $\times \;10^{-18}$	1.09 $\times \;10^{-17}$	3.20 $\times \;10^{-18}$
F29	7.64 $\times \;10^{-18}$	2.00 $\times \;10^{-8}$	4.09 $\times \;10^{-18}$	7.21 $\times \;10^{-18}$	3.36 $\times \;10^{-18}$
F30	3.01 $\times \;10^{-18}$	3.01 $\times \;10^{-18}$	3.25 $\times \;10^{-18}$	3.20 $\times \;10^{-18}$	3.20 $\times \;10^{-18}$
+/=/−	29/0/0	27/0/2	27/0/2	26/0/3	24/0/5

**Table 10:** Error evaluation of different models.

Prediction Model	MAPE	RMSE	MAE	$R^{2}$
BP	0.1708	32.7891	51.1441	0.8775
LSTM	0.2167	32.9238	38.1506	0.9318
WOA-LSTM	0.1996	25.4375	28.2801	0.9627
GWO-LSTM	0.1651	27.4063	33.6791	0.9469
PSO-LSTM	0.1765	24.2636	29.6215	0.9589
SSA-LSTM	0.1755	23.7631	27.4359	0.9647
MSSA-LSTM	0.1374	23.6229	26.5938	0.9668
ASDSSA-LSTM	0.1247	19.4063	21.8124	0.9777

## Data Availability

Not applicable.

## References

[1] Kumar V., Chhabra J.K., Kumar D. (2014). Parameter adaptive harmony search algorithm for unimodal and multimodal optimization problems. J. Comput. Sci..

[2] Mohamed A.W., Hadi A.A., Mohamed A.K. (2020). Gaining-sharing knowledge based algorithm for solving optimization problems: A novel nature-inspired algorithm. Int. J. Mach. Learn. Cyber..

[3] Holland J.H. (1992). Genetic algorithms. Sci. Am..

[4] Storn J.R., Price K. (1997). Differential evolution—A simple and efficient heuristic for global optimization over continuous spaces. J. Glob. Optim..

[5] Beyer H.G., Schwefel H.P. (2002). Evolution strategies—A comprehensive introduction. Nat. Computing..

[6] Glover F. (1989). Tabu search—Part I. ORSA J. Comput..

[7] Kirkpatrick S., Gelatt C.D., Vecchi M.P. (1983). Optimization by simulated annealing. Science.

[8] Kennedy J., Eberhart R. Particle swarm optimization. Proceedings of the IEEE International Conference on Neural Networks.

[9] Yang X.S., Deb S. (2010). Cuckoo Search via Lévy flights. World Congr. Nat. Biol. Inspired Comput..

[10] Rashedi E., Nezamabadi-Pour H., Saryazdi S. (2009). GSA: A gravitational search algorithm. Inf. Sci..

[11] Formato R.A. (2007). Central force optimization: A new meta-heuristic with applications in applied electromagnetics. Prog. Electromagn. Res..

[12] Krishnanand K.N., Ghose D. Detection of multiple source locations using a glowworm metaphor with applications to collective robotics. Proceedings of the 2005 IEEE Swarm Intelligence Symposium.

[13] Pan W.T. (2012). A new fruit fly optimization algorithm: Taking the financial distress model as an example. Knowl.-Based Syst..

[14] Liu C.Y., Yan X.H., Wu H. (2011). The wolf colony algorithm and its application. Chin. J. Electron..

[15] Tang R., Fong S., Yang X.S. Wolf search algorithm with ephemeral memory. Proceedings of the Seventh International Conference on Digital Information Management (ICDIM 2012).

[16] Fong S., Deb S., Yang X.S. (2015). A heuristic optimization method inspired by wolf preying behavior. Neural Comput. Appl..

[17] Mirjalili S., Mirjalili S.M., Lewis A. (2014). Grey wolf optimizer. Adv. Eng. Softw..

[18] Meng X., Liu Y., Gao X. (2008). A new bio-inspired algorithm: Chicken swarm optimization. J. Abbr..

[19] Uymaz S.A., Tezel G., Yel E. (2015). Artificial algae algorithm (AAA) for nonlinear global optimization. Appl. Soft Comput..

[20] Mirjalili S. (2015). The ant lion optimizer. Adv. Eng. Softw..

[21] Seyedali M., Andrew L. (2016). The whale optimization algorithm. Adv. Eng. Softw..

[22] Mashwani W.K., Salhi A., Jan M.A. (2016). Evolutionary Algorithms Based on Decomposition and Indicator Functions: State-of-the-art Survey. Int. J. Adv. Comput. Sci. Appl..

[23] Mirjalili S., Gandomi A.H., Mirjalili S.Z. (2017). Salp Swarm Algorithm: A bio-inspired optimizer for engineering design problems. Adv. Eng. Softw..

[24] Arora S., Singh S., Yetilmezsoy K. (2018). A modified butterfly optimization algorithm for mechanical design optimization problems. J. Braz. Soc. Mech. Sci. Eng..

[25] Xue J., Shen B. (2020). A novel swarm intelligence optimization approach: Sparrow search algorithm. Syst. Sci. Control Eng..

[26] Mashwani W.K., Haider R., Belhaouari S.B. (2021). A Multiswarm Intelligence Algorithm for Expensive Bound Constrained Optimization Problems. Complexity..

[27] Mashwani W.K., Shah H., Kaur M. (2021). Large-scale bound-constrained optimization based on the hybrid teaching-learning optimization algorithm. Alex. Eng. J..

[28] Rao R.V., Savsani V.J., Vakharia D.P. (2021). Teaching—Learning-based optimization: A novel method for constrained mechanical design optimization problems. Comput.-Aided Des..

[29] Mashwani W.K., Shah S., Belhaouari S.B. (2021). Ameliorated Ensemble Strategy Based Evolutionary Algorithm with Dynamic Resources Allocations. Int. J. Comput. Intell. Syst..

[30] Rodríguez-Ramos A., Bernal-de-Lázaro J.M., Neto A.J.S. (2019). Fault Detection Using Kernel Computational Intelligence Algorithms. Computational Intelligence, Optimization and Inverse Problems with Applications in Engineering.

[31] Yang X.S., Gandomi A.H. (2012). Bat Algorithm: A Novel Approach for Global Engineering Optimization. Eng. Comput..

[32] Liu T., Yuan Z., Wu L. (2021). An optimal brain tumor detection by convolutional neural network and Enhanced Sparrow Search Algorithm. Proc. Inst. Mech. Eng. Part H J. Eng. Med..

[33] Liu G.Y., Shu C., Liang Z.W. (2021). A modified sparrow search algorithm with application in 3d route planning for UAV. Sensors.

[34] Wang P., Zhang Y., Yang H. (2021). Research on economic optimization of microgrid cluster based on chaos sparrow search algorithm. Comput. Intell. Neurosci..

[35] Tang Y., Li C., Li S. (2021). A fusion crossover mutation sparrow search algorithm. Math. Probl. Eng..

[36] Chen X., Huang X., Zhu D. (2021). Research on chaotic flying sparrow search algorithm. J. Phys. Conf. Ser..

[37] Ouyang C., Qiu Y., Zhu D. A multi-strategy improved sparrow search algorithm. Proceedings of the 2021 4th International Conference on Advanced Algorithms and Control Engineering (ICAACE 2021).

[38] Zhang C., Ding S. (2021). A stochastic configuration network based on chaotic sparrow search algorithm. Knowl.-Based Syst..

[39] Yuan J., Zhao Z., Liu Y. (2021). DMPPT Control of Photovoltaic Microgrid Based on Improved Sparrow Search Algorithm. IEEE Access.

[40] Zhu Y., Yousefi N. (2021). Optimal parameter identification of PEMFC stacks using Adaptive Sparrow Search Algorithm. Int. J. Hydrog. Energy.

[41] Zhang J., Xia K., He Z. (2021). Semi-supervised ensemble classifier with improved sparrow search algorithm and its application in pulmonary nodule detection. Math. Probl. Eng..

[42] Mao Q.H., Zhang Q. (2021). Improved Sparrow Algorithm Combining Cauchy Mutation and Opposition-Based Learning. J. Front. Comput. Sci. Technol..

[43] Fu H., Liu H. (2022). Improved sparrow search algorithm with multi-strategy integration and its application. Control. Decis..

[44] Arora S., Anand P. (2019). Chaotic grasshopper optimization algorithm for global optimization. Neural Comput. Appl..

[45] Yanling W. Image Scrambling Method Based on Chaotic Sequences and Mapping. Proceedings of the 2009 First International Workshop on Education Technology and Computer Science.

[46] Tunay M., Abiyev R. (2022). Improved Hypercube Optimisation Search Algorithm for Optimisation of High Dimensional Functions. Math. Probl. Eng..

[47] Simon D. (2008). Biogeography-based optimization. IEEE Trans. Evol. Comput..

[48] Yao X., Liu Y., Lin G. (1999). Evolutionary programming made faster. IEEE Trans. Evol. Comput..

[49] Stanovov V., Akhmedova S., Semenkin E. LSHADE algorithm with rank-based selective pressure strategy for solving CEC 2017 benchmark problems. Proceedings of the 2018 IEEE congress on evolutionary computation (CEC).

[50] Tanabe R., Fukunaga A.S. (2014). Improving the search performance of SHADE using linear population size reduction. IEEE Congress on Evolutionary Computation (CEC).

[51] Derrac J., García S., Molina D., Herrera F. (2011). A practical tutorial on the use of nonparametric statistical tests as a methodology for comparing evolutionary and swarm intelligence algorithms. Swarm Evol. Comput..

[52] Smith B.L., Demetsky M.J. (1997). Traffic Flow Forecasting: Comparison of Modeling Approaches. J. Transp. Eng..

[53] Wei Y., Chen M.C. (2012). Forecasting the short-term metro passenger flow with empirical mode decomposition and neural networks. Transp. Res. Part C Emerg. Technol..

[54] Duan Y.J., Lv Y.S., Zhang J. (2016). Deep learning for control: The state of the art and prospects. Acta Autom. Sin..

